# Optimizing predictive performance of criminal recidivism models using registration data with binary and survival outcomes

**DOI:** 10.1371/journal.pone.0213245

**Published:** 2019-03-08

**Authors:** Nikolaj Tollenaar, Peter G. M. van der Heijden

**Affiliations:** 1 Research and Documentation Centre (WODC), Ministry of Justice and Security, The Hague, Zuid-Holland, the Netherlands; 2 Department of Social Sciences, Utrecht University, Utrecht, Utrecht, the Netherlands; 3 Department of Social Sciences, University of Southampton, Hampshire, United Kingdom; University of Maribor, SLOVENIA

## Abstract

In a recidivism prediction context, there is no consensus on which modeling strategy should be followed for obtaining an optimal prediction model. In previous papers, a range of statistical and machine learning techniques were benchmarked on recidivism data with a binary outcome. However, two important tree ensemble methods, namely gradient boosting and random forests were not extensively evaluated. In this paper, we further explore the modeling potential of these techniques in the binary outcome criminal prediction context. Additionally, we explore the predictive potential of classical statistical and machine learning methods for censored time-to-event data. A range of statistical manually specified statistical and (semi-)automatic machine learning models is fitted on Dutch recidivism data, both for the binary outcome case and censored outcome case. To enhance generalizability of results, the same models are applied to two historical American data sets, the North Carolina prison data. For all datasets, (semi-) automatic modeling in the binary case seems to provide no improvement over an appropriately manually specified traditional statistical model. There is however evidence of slightly improved performance of gradient boosting in survival data. Results on the reconviction data from two sources suggest that both statistical and machine learning should be tried out for obtaining an optimal model. Even if a flexible black-box model does not improve upon the predictions of a manually specified model, it can serve as a test whether important interactions are missing or other misspecification of the model are present and can thus provide more security in the modeling process.

## Introduction

Prediction models have many different applications in the field of criminal justice. These models can be used on a group level or on an individual level. On a group level, prediction models can be used to estimate effects of interventions on groups of offenders. When control group data are unavailable, a population prediction model can provide the expected recidivism when no effect was to be expected (see e.g. [1]). Group level prediction models can be used to adjust recidivism trends for fluctuations due to shifts in the characteristics of the offender population, in order to monitor criminal justice policies regarding recidivism reduction.

On the individual level, the typical application of prediction models is the development of risk assessment scales, used to construct risk groups, create orderings on recidivism risk or to classify offenders (i.e. a decision instrument). Risk assessment scales are commonly applied in various stages of criminal proceedings, to efficiently allocate either repressive or treatment resources to offenders. In the first case, scales can support decisions regarding incarceration duration, parole or intensive supervision based on an estimated future risk. In the second case, high risk individuals are allocated to treatments that mitigate recidivism risk are as they would most likely benefit most from treatment in terms of recidivism.

For decades, researchers have tried to improve prediction scales by searching for strong indicators that predict different kinds of recidivism, predominantly for violent and sexual recidivism [2–7]. In order to obtain an optimal composite risk score, the variables should not be equally weighted. Therefore, scale weights are usually estimated using a statistical model for binary outcomes, logistic regression. Logistic regression implies that the data are generated from a probability model, i.e., the underlying outcome is Bernoulli distributed and linearly related to independent variables on the logit scale. The unique solution to the regression weights is obtained at the minimum of the negative log-likelihood. The resulting weights should generate an optimal prediction given the available data, as follows from the inferential statistical model.

Pure prediction is however an endeavor entirely different from explanation or statistical inference. This is not always explicitly stated in statistics methodology literature, and requires a different strategy [8]. When applying statistical models for prediction, the standard textbook advice on statistical modeling should not be followed (see the reaction of Efron, in [9]). For the goal of prediction, one does not require a simple parsimonious model, a ‘correct’ model that only includes variables backed by theory and does not miss important causally related variables, or a data set that does not violate the assumptions of the model. The ultimate utility of a prediction model is in how well it predicts empirically on unseen data.

This standpoint has been exploited in computer science with the aid of increasingly faster and high-memory computers since the 1980s, the emergence of large databases with many non-theoretical variables and various flexible non-statistical algorithmic classification approaches to modeling were developed that rely less or not at all on assuming a certain data generating mechanism [10], nowadays usually termed machine learning. These techniques, unlike statistical models, take a data centric approach and are more engineering oriented rather than science oriented. Instead of modeling the probability of a presupposed underlying stochastic model, typically an adaptive flexible function is fitted that approaches the observed outcomes well. Model fitting is typically done by minimizing a loss function that measures the discrepancy between the observed and predicted values directly instead of using theoretical maximum likelihood based statistical criteria.

These techniques proved to have several advantages over the classical statistical algorithms in the context of prediction. Many of the algorithms automatically handle nonlinear relations with the outcome as well as find interaction effects, although sometimes transforming the input data (i.e. feature engineering and feature selection are required for optimal performance. Additionally, these algorithmic approaches should work well in situations in which standard statistical modeling typically fails, like with noisy data, data with many correlated predictors or many irrelevant predictors. Of course, there are disadvantages too. For each new sample, manual tuning is needed by the researcher in order for the model to function optimally, which can be tedious for multiple (continuous) tuning parameters. When using large data, a lot of computation time and working memory are needed for estimating many of these models. Furthermore, many machine learning algorithms are more sensitive to class imbalance (i.e. a skewed distribution of the binary outcome) than statistical algorithms. Solutions to this problem as proposed in the machine learning community (see e.g. [11] for an overview) are not always generally applicable or effective. Additionally, individual probabilities of popular machine learning algorithms can differ from run to run with the same model specification but with a different seed for the random number generator. This property renders decision making on individual probabilities questionable. For instance in random forests, stochastic gradient boosting and bagging, variance in the individual probabilities are caused by the sampling incorporated in the algorithm. This variance can however be decreased by using a relatively large number of trees or increasing the amount of data. In overparameterized models like neural networks, various weight configurations with equal minimal loss can be found when using different random starting values, or the algorithm can get stuck in one of the local minima of the nonconvex loss function. Therefore, also for this model class, different runs will generate different probabilities for individuals, which can result in different risk categories or different classification outcomes due to inherent randomness. However, for group level prediction models, this is an unproblematic property, as observation level variance is averaged out and group scores will be equal.

The difference between more traditional (statistical) models and machine learning models are mainly concentrated on how nonlinearities and interactions are dealt with. In traditional modeling, nonlinearity can be assessed by the researcher in an exploratory data analysis phase of modeling for each covariate (see also [8]), appropriate transformations like those from the Tukey power family [12] can be applied to the predictors, and then the transformed predictor can be incorporated explicitly in a linear model. A more flexible nonparametric statistical approach entails each continuous predictor having its own degrees of freedom of nonlinearity or a ‘roughness’ penalty [13]. Variable interactions are mostly dealt with by explicitly including hand picked multiplicative interactions.

Because the main difference of traditional statistics and machine learning is in how nonlinearity and interactions are dealt with, machine learning is expected to improve models in terms of predictive performance when complex interactions and hard to explicitly or nonparametrically model nonlinearities in the data of interest empirically exist in the data.

### Comparative predictive performance

Machine learning algorithms have proved to work well for many different practical prediction problems. The promise of improved model accuracy and substantial monetary rewards in business and competitions (e.g. the Kaggle competitions, the KDD cup and the Netflix competition) have made this a very active research field. Driven by experimentation to optimize performance, it is yet not fully understood why and when many of these algorithms work well (see e.g. [14]). Neither is it clear in advance which algorithm works best on what type of data. Therefore, many advise to compare many different models on each new data set. There are however models that should perform well on a wide range of data sets, such as boosting, random forests and linear discriminant analysis. This notion of a good overall model has led to many algorithm comparison studies in the field of machine learning (see e.g. [15–17]).

There are, however, several problems that may limit the generalizability of the results found in these empirical classifier comparison studies. First, many of these studies are performed on different selections of data sets from the University of Carolina Irvine (UCI) machine learning repository [18]. This online repository contains classification data sets ranging widely from computer vision, text classification and speech recognition data to medical diagnosis and astronomical data, but as of yet it does not contain criminological data. According to [19], there is some selectivity in the UCI data base. A lack of big and difficult to classify data sets is perceived with a surplus of ‘too easy’ small data sets, which may lead to a form of publication bias in the results of classifier comparisons. The UCI database is intensively used for evaluating (relative) classifier performance to this day.

Secondly, machine learning comparison studies that include classical statistical models like linear discriminant analysis or logistic regression, do not mention applying linearizing transformations prior to fitting the model where appropriate, while this is common practice in statistical modeling. When the data contain predictors measured at the interval level, leaving continuous predictors untransformed in a linear model will give intrinsically nonlinear algorithms an unfair advantage. The implicit actual comparison in this ‘naïve researcher scenario’ is which *automatic* model has the best predictive performance. In traditional statistical modeling, failure to account for nonlinearity is a form of model misspecification. Unfortunately many examples of naïve statistical modeling can be identified in the criminological (prediction) literature.

There are also studies that use simulated data to assess the relative performance of machine learning algorithms (see e.g. [20]). Although these are insightful as to how well behaved the different algorithms are, generalizing results from simulated synthetic data to actual data collected in real life is hard. Moreover, as the data generating distribution is known, it is difficult to create a realistic comparison scenario for statistical models, as a correctly specified model will almost always outperform a nonparametric approximation.

Results from machine learning studies may not be representative to the criminal justice applications of recidivism risk prediction. Therefore, regardless of the flaws in comparison studies, in our opinion it is better to instead form an empirical judgment about the applicability of algorithms on data from the domain of interest, in our case the criminal justice domain.

In recent years, algorithmic modeling is increasingly being tried out for predicting different sorts of recidivism risk [21–30], predicting court decisions [31] and predicting prison misconduct [32]. The majority of these studies focuses on classification models, i.e. predicting a yes/no response. With [24], the discussion of the merits of flexible (machine learning) modeling over classical statistical modeling surfaced in the field of criminology. It is argued that previous comparison studies did not include two state-of-the art methods that would have shown superiority in performance, namely random forests [33] and stochastic gradient boosting [34]. Using an artificial data example having two decision boundaries instead of one, they demonstrate that logistic regression fails and the tree-based ensemble methods gradient boosting machine and random forest obtain high predictive accuracy. In a real data set they show that the two tree-based ensemble methods outperform logistic regression, where again no mention is made of applying linearizing transformations or quadratic terms before fitting the logistic regression. By applying these transformations, a nonlinear decision boundary is essentially made linear again. This absence of the linearizing transformations when logistic regression is compared to a machine learning model on data having continuous predictors is unfortunately very common in criminological comparison studies, as in [22–23, 25, 28–29, 35] or restricted to quadratic terms [36]. This absence of linearizing transformations applies to the majority of machine learning comparison studies that include linear statistical methods.

It is however crucial to know whether linearizing transformations or categorization of continuous variables were applied, because for instance the relation of number of previous convictions to recidivism levels off as the number of convictions increases (see e.g. [37]), while criminal history is the strongest predictor of recidivism (see e.g. [3]). Additionally, the effect of age on reconviction is often more adequately modeled nonlinearly by including age squared. Thus, a significant portion of the reported performance disparity is highly likely to be due to statistical model misspecification and not to the statistical model itself.

So, exploratively applying and checking for a handful of canonical variable transformations (i.e. log(*x*), 1/*x*, √*x*, *x*^2^ or higher order polynomial) on the estimation portion of a data set can linearize many datasets very effectively, making it possible to use linear models again. Moreover, this strategy retains several advantages of linear statistical models, namely transparency, interpretability, requiring less data, a unique solution for individual probabilities, low variance estimates and fast estimation.

The discussion of machine learning performance is mostly limited to binary outcome classification models. Performance results of binary outcome studies therefore only apply to models in which a fixed time point of the event of interest (e.g. recidivism at two years) is chosen. However, in some situations, not all observations are observed up to the time point of interest (i.e. right censored data), or one also needs probability estimates on intermediate time points, or the median time to recidivism is of interest. For these cases, variants of survival analysis are useful. A range of popular machine learning algorithms has already been generalized to right censored (survival) data [38–42]. At the same time, the methodology of evaluating the fit and performance of survival models has improved considerably in the last fifteen years. Moreover, software for state-of-the-art techniques for modeling and establishing prediction error has become available due to the increasing adoption of the R statistical programming language [43].

In survival analysis, at each time point the observed value is only known for uncensored observations. With the invention of the Inverse Probability of Censoring Weights (IPCW) estimator [44–45] it became possible to generalize binary criterion measures to censored data at arbitrary time points. By using this weight estimator, bias by partly censored data is circumvented by reweighting the uncensored observations, thereby making it possible to make unbiased predictions of statistics using only the uncensored observations. This makes model comparisons in survival data feasible on statistics that depend on the actually observed outcome. [46] devised the time dependent ROC curve, a time dependent version of the *C*-index (a.k.a. concordance, [47]) which is similar to the area under the ROC curve (AUC, [48]). [49] developed the integrated Brier score as a criterion for model fit. This is a criterion for survival data that measures accuracy but also has an element of calibration. All these statistics of fit can be used for every conceivable survival model that can generate cumulative survival probabilities.

Since the field of machine learning for censored data is relatively young and publicly available software for these methods has only recently become available, to our knowledge there are virtually no systematic large scale comparative studies of machine learning models for censored survival data. The comparisons that have been made mainly come from the biomedical literature, where they are mainly restricted to neural network models and survival trees. For instance [50–51] compared Cox regression to several types of neural network models on simulated censored data. The first study found mixed results, whereas in the second study, Cox regression was never outperformed if correctly specified. [52] compared different approaches of survival tree models. [53] compared Cox regression to neural networks on breast cancer data and found neural networks to work better. However, they did not do any data transformations for continuous variables before fitting the Cox regression. [54] compared different versions of survival support vector machines (SVM) with Cox regression. They found no significant improvement of survival-SVM over Cox.

This study has two objectives. First, we re-analyze the data of [30] to see whether the random forests, stochastic gradient boosting and Bayesian additive regression trees techniques as indicated by [24] offer improved prediction in actual recidivism data of three types of recidivism. Effectively, we test whether improvements in predictive performance can be attained over a adequately specified statistical model by using machine learning methods. Additionally, we evaluate penalized logistic regression and penalized linear discriminant analysis (see e.g. [55]) to see whether coefficient shrinkage can improve these classical methods. In order to see whether these results generalize beyond these data, we also fit the models on the North Carolina prison data [56].

Secondly, as little is known about the predictive performance of survival analysis variants of machine learning in survival data, machine learning survival analysis variants will be compared to the conventional statistical types of survival analysis, namely Cox regression, parametric survival models, Aalen regression and split population Cox models.

Within the comparison of this study, being human specified statistical models compared to different machine learning models, the expected gains in predictive performance will be dependent on the empirical existence of complex interactions and nonlinearities in the data, that are hard to model explicitly.

## Method

### Data used

#### Dutch offender’s index data

The data for the binary models are the same as in [30]. They consist of all adult perpetrators from the Dutch offender’s index (DOI) found guilty during criminal proceedings ending in 2005. The DOI is an automated, encrypted and anonymized version of the Judicial Information System (JDS) that probation officers and others can request for an offender. The DOI provides a chronological overview of all criminal cases in which a physical or legal entity has been suspected of a criminal offence. Criminal cases are registered for persons from ages 12 and up, as the minimal age of criminal responsibility is 12 years.

For general recidivism, a sample of 20,000 of the 2005 year was taken, whereas for violent recidivism, a sample of 20,000 observations convicted for violence was taken. The sexual recidivism data consisted of all sexual offenders of 2005 and a part of the sexual offenders of 2006 (*N* = 1,332). The general and the violent sample were split into 10,000 observations as estimation data, whereas the other 10,000 were kept as a separate test set. For the sexual recidivism, 70% of the data formed the estimation set (*n* = 932), whereas thirty percent of the data was kept as the separate test set (*n* = 400). In all data, within the cohort year, the first case was selected as the index case, so every subject will appear only once in each separate selection. For further details concerning the data, we refer to [30].

For the survival data, we used the 2006/2007 data of the DOI. We used 20,000 observations for general recidivism (2006; 44.8% were found to recidivate), 20,000 for violent recidivism (2006; 28.7% were found to recidivate) and all sexual offenders (2006/2007; *N* = 2,058, 4.1% were found to recidivate). The data were right censored at the 10^th^ of July, 2012. We used the same data splitting scheme as for the binary outcome data above, except that the sexual survival data were split 50/50. The background characteristics of the samples are shown in Table 1.

**Table 1:** Sample characteristics 2006* DOI data.

	General recidivism	Violent recidivism	Sexual recidivism
Total *n*	20,000	20,000	2,058
average observation time in months	58.8	71.6	80.7
% experiencing event	44.8	28.7	4.1
4 year base rate (%)	37.7	22.6	3.1
Gender: female(%)	15.6	10.3	-
Age in years (mean)	35.8	34.8	39.5
Age of first conviction (mean)	27.9	25.5	31.2
Most serious offence type (%)			
Violence	14.5	100.0	0.5
Sexual	0.7	0.5	97.6
Property with violence	1.2	1.2	0.8
Property without violence	22.7	4.2	0
Public order	10.3	13.8	0
Drug offence	6.9	1.0	0
Motoring offence	30.8	1.5	0
Misc. offence	13.0	6.3	1.0
Country of birth (%)			
Netherlands	71.7	72.0	73.6
Morocco	2.8	3.9	2.7
Neth. Antilles/Aruba	2.7	3.5	3.2
Surinam	4.5	5.0	4.5
Turkey	3.0	3.7	2.2
Other Western countries	8.1	4.9	5.6
Other non-Western countries	7.2	7.0	8.2
Offence type present in index case (%)			
Violence component (0/1)	15.9	100.0	12.6
Sexual component	0.7	0.5	100.0
Property with violence	1.2	1.2	1.7
Property without violence	23.3	4.2	3.1
Public order	13.0	13.8	6.2
Drug offence	8.2	1.0	0.9
Motoring offence	31.5	1.5	0.3
Misc. offence	15.1	6.3	9.4
Criminal history counts (mean)			
Conviction density	0.4	0.5	0.3
Number of previous convictions	4.4	5.4	3.7
Previous violent offences	0.5	1.0	0.5
Previous sexual offences	0.0	0.0	0.2
Previous property with			
violence offences	0.1	0.2	0.2
Previous property offences	2.3	2.4	1.5
Previous public order offences	0.6	1.0	0.6
Previous drug offences	0.2	0.2	0.1
Previous motoring offences	0.8	0.8	0.6
Previous misc. offences	0.3	0.4	0.3
Previous prison terms	0.9	1.0	0.7
Previous community service orders	0.3	0.5	0.3
Previous fines	0.9	1.2	0.8
Previous PPDs^†^	0.5	0.5	0.3

Note: The sexual recidivism data also contain data from 2007, to enlarge the sample size.

^†^Public prosecutor's disposals.

#### North Carolina prison data

In order to investigate the generalizability of the results, we also included the public access data sets used by [56], the North Carolina prison data. These data consist of all individuals released from prison from 1 July 1977 through 30 June 1978 (the 1978 cohort) and from 1 July 1979 through 30 June 1980 (the 1980 cohort). Both cohorts were randomly split by Schmidt and Witte into estimation and validation samples. Originally used to compare survival analysis models, these data are also suitable for binary outcome analyses as all observations have a follow-up time longer than four years. Apart from the recidivism variables, the data contain 15 background characteristics: time served (in months) for the sample sentence (TSERVD); age in months (AGE); number of previous incarcerations (PRIORS); number of prison rule violations in sample sentence (RULE); number of years of formal schooling completed at prison entry date of sample sentence (SCHOOL); a dummy for being non-black (WHITE); gender (MALE); dummy for serious alcohol problems in record (ALCHY); dummy for indication of hard drug use in record (JUNKY); dummy for being married at prison entry date of sample sentence (MARRIED); dummy for supervised release (e.g. parole) following sample sentence (SUPER); dummy for participation in North Carolina prisoner work release program during sample sentence (WORKREL); dummy for felony/misdemeanor (felony = 1) in sample sentence (FELON); dummy for crime against a person in sample sentence (PERSON); dummy for crime against property in sample sentence (PROPTY).

Table 2 shows the background characteristics of all samples.

**Table 2:** Sample characteristics North Carolina prison data.

	1978		1980	
	Estimation	Validation	Estimation	Validation
	*N* = 1,540	*N* = 3,078	*N* = 1,435	*N* = 4,304
Observation time in months (mean)	56.0	56.1	39.4	38.7
% experiencing event	37.0	37.4	37.0	37.5
4 year base rate (%)	32.1	32.3	36.1	37.1
TSERVD (mean)	18.8	19.8	19.5	19.2
AGE (mean)	346.1	342.3	339.0	341.9
PRIORS (mean)	1.4	1.4	1.4	1.3
RULE (mean)	1.1	1.3	1.5	1.5
SCHOOL (mean)	9.7	9.7	9.6	9.6
WHITE (%)	50.9	52.2	51.0	51.1
MALE (%)	93.8	94.3	94.6	94.4
ALCHY (%)	21.0	19.6	35.7	36.0
JUNKY (%)	23.9	27.2	21.8	19.6
MARRIED (%)	25.6	26.8	23.4	23.3
SUPER (%)	69.8	69.6	80.1	81.6
WORKREL (%)	45.5	46.5	43.3	42.9
FELON (%)	31.2	32.1	43.1	41.3
PERSON (%)	6.0	5.3	11.3	11.2
PROPTY (%)	25.1	25.7	44.7	44.0

For details with regard to the data, we refer to [56] (p. 21 and further). We tested both cohorts, because the 1980 cohort is notably harder to predict than the 1978 cohort.

### Models used

The sets of methods used are different for the binary case and the censored data case, because for survival analysis not all methods have been generalized yet or are readily available in statistical software.

#### Binary case

In this paper, some of the models used are different from those in [30]. For the results on MARS, FDA, adaBoost and linear kernel SVM, we refer to that paper. In the comparison of models on a binary outcome of four year recidivism (yes/no), the models used and their respective tuning parameter values are depicted in Table 3.

**Table 3:** Used models and tuning parameter values used for the binary outcome data.

	Parameter 1	Parameter 2	Parameter 3	Parameter 4
Linear logistic regression [57]	-	-	-	-
Linear discriminant analysis [58]	-	-	-	-
*L*_1_-logistic regression^†^ [55, 59–60]	λ_1_ = 0.00001, 0.0001, 0.001, 0.01, 0.1, 0.5, 1, 10, 20, 30, 50, 70, 100, 500, 1,000	-	-	-
*L*_2_-logistic regression^†^ [55, 61–62]	λ_2_ = 0.00001, 0.0001, 0.001, 0.01, 0.1, 0.5, 1, 10, 20, 30, 50, 70, 100, 500, 1,000	-	-	-
Penalized discriminant analysis^†^ [63]	λ = 0.00001, 0.0001, 0.001, 0.01, 0.1, 0.5, 1, 10, 20, 30, 50, 70, 100, 500, 1,000	-	-	-
Random forest [33]	N_trees_: 1,000	N_predictors_: 2, 3, 4, 5, 6, 7, 9, 11, 13, 16	Node size = 1	-
Stochastic gradient boosting [34]	Max. N_trees_: 1,000	Interaction depth: 2, 3, 4, 5, 6, 7, 8, 9	Bag fraction = 0.5	*ν* = 0.01
BART [64–65]	N_trees_: 200	*k* = 2.0	N_iter_:1,000	Number of burn-in iterations: 100

^†^ Models were tried out both with standardized and unstandardized input data.

#### Survival case

In the comparison of models on a survival outcome, we used the models and their respective tuning parameter values as shown in Table 4.

**Table 4:** Tuning parameters used for the survival data.

	Parameter 1	Parameter2	Parameter 3
Cox regression [66].	-	-	-
Aalen regression^†^ [67–68].	-	-	-
Cox-cure model‡ [69–70]	-	-	-
Parametric survival models (Exponential, Weibull, Log-logistic, Lognormal)[71]	-	-	-
Random survival forest [40]	N_trees_: 1,000	N_predictors_: 2, 3, 4, 5, 6, 7, 9, 11, 13, 16	Node size = 3
Ridge Cox boosting^§^ [72]	Max. number of iterations: 1,000	*ν* = 0.1	c_smf_ = 1
Stochastic gradient boosting survival analysis [73]	N_iter_: 1,000 optimal N_iter_ by 10-fold cv	Interaction depth: 2, 3, 4, 5, 6, 7, 8, 9	Bag fraction = .5
*L*_1_-Cox regression^§^ [74]	λ_1_ = 0.00001, 0.0001, 0.001, 0.01, 0.1, 0.5, 1, 10, 20, 30, 50, 70, 100, 500, 1,000	-	-
*L*_2_-Cox regression^§^ [75]	λ_2_ = 0.00001, 0.0001, 0.001, 0.01, 0.1, 0.5, 1, 10, 20, 30, 50, 70, 100, 500, 1,000	-	-
Single hidden layer Cox neural network^ǁ^ [42, 76]	Size of hidden layer = 1, 3, 5, 7, 9, 11, 13, 15, 17, 19	Weight decay = 0, 0.1, 0.01, 0.001, 0.0001	-
Single hidden layer parametric Neural network models^ǁ^ [42, 76] (Exponential, Weibull, Log-logistic, Lognormal)	Size of hidden layer = 1, 3, 5, 7, 9, 11, 13, 15, 17, 19	Weight decay = 0, 0.1, 0.01, 0.001, 0.0001	-
PLS^§^ [77, 78]	N_components_: 2, 3, 4, 5, 6, 7, 8, 9, 10	-	-

^†^Fully non-parametric Aalen models were used, as opposed to semi-parametric Cox-Aalen models [79].

^‡^The cure parameter was specified to depend on the same variables as the Cox part of the model.

§ Models were tried out both with standardized and unstandardized input data.

^ǁ^ Prior to modeling, the data were rescaled between 0–1 to equate the influence of weight decay on each covariate. The weight decay is identical to the λ in *L*_2_*-*Cox-regression.

### Software used

All analyses were performed using R 2.15.3, using a combination of CRAN libraries (see S1 File for the complete list) and custom written R-code. In the circumstance that an individual analysis caused R to crash, we instead used the Python programming language version 2.7.6 in combination with the scikit-learn machine learning library [80] version 0.15.1. This was necessary for both North Carolina Prison data sets using gradient boosting.

### Performance metrics used for comparison of models and methods

We require different metrics for predictive performance because recidivism prediction models can be used for three different purposes. These purposes are: the *ordering* of risk scores (discrimination), the estimation of an exact *probability* of recidivism (calibration/reliability, or the forming of risk groups) and the *classification* of offenders into recidivists/non-recidivists (´clinical´ usefulness). For these purposes, we used a range of different metrics of performance. Therefore, instead of relying on a single measure, models were compared on a range of metrics that each emphasize these different aspects of model quality on these purposes.

### Performance metrics for binary outcome data

The methodology for evaluating model quality is well developed for the binary case and has resulted in a multitude of performance metrics. The wide range of metrics used ensures that every aspect of prediction model performance is covered.

Area Under the ROC Curve (AUC). The AUC (Area under the ROC curve, [48]) is the widely used quantification of discrimination. The value is the proportion of all possible positive-negative pairs that are ordered correctly on the risk score.

H-Statistic ([81]). The H-statistic is an improvement of the AUC. [81] discovered that the AUC is incoherent, when it is interpreted as classification accuracy aggregated over thresholds. He proved that it treats the relative severities of false positives and false negatives differently for different classifiers. The H-measure lets the researcher fix the distribution of relative severities to make it classifier independent. The measure was later improved in [82]. This is the version we calculated in this paper. We used the default severity ratio of the observed proportions of observed recidivists and observed non-recidivists in the data.

Accuracy (ACC). The accuracy is the sum of true positives and true negatives divided by the total number of instances, in other words the percentage classified correctly. In this paper, it is evaluated at two cutoffs, namely 0.5 and the base rate.

Root mean squared error (RMSE). A well-known measure of divergence of predictions and actual values in linear regression. It is defined as:

$$\sqrt{\sum_{i=1}^{n}\frac{{\left(y_{i}-{\hat{y}}_{i}\right)}^{2}}{n}}$$

where y_i_ are the observed outcomes, ${\hat{y}}_{i}$ are the predicted outcomes and *n* is the total number of observations. The RMSE is also applicable in the binary outcome case, in which the predictions ${\hat{y}}_{i}$ are the predicted probabilities.

SAR ([83]). A combination measure of RMSE, AUC and ACC, given by:

$$\frac{(1-RMSE)+AUC+ACC}{3}$$

The three criteria in this measure were empirically found to diverge most often and to correlate highest with other possible fit criteria. The SAR was therefore proposed to have one robust measure to establish optimality in different domains.

CAL (calibration error [83]). This is the average error when calculating the difference between the moving average of the predicted probability and the corresponding observed proportion of positive outcomes. A window of 100 observations is used.

ACC(SENS = SPEC). Accuracy when sensitivity equals specificity (see [30]). This is a measure of accuracy which is independent of variations in the amount of calibration of the risk scores over different models and a specific cutoff-score. It gives the accuracy when false positives are weighted equally severely as false negatives. This metric can be useful in low base-rate data and is independent from how well the probabilities are calibrated.

#### Performance metrics for survival data

The methodology for evaluating model quality in survival regression on censored data was long restricted to various types of residual analysis [84–86] and specific goodness-of-fit tests for the Cox regression model or other specific models (e.g. [87]). Only more recently, the notion of prediction error for survival models in general was worked out. In this methodology, it does not matter how the survival function is estimated, either semi-parametrically or parametrically.

Integrated Brier score (IBS [49]). This is a measure that has elements of both calibration and accuracy. It is analogous to the Brier score known from the binary case [88], the Brier score (BS) in the survival context at a specific time *t* is the average of the sum of squared differences between the observed value (1 = event, 0 = censored or event-free) and the predicted cumulative survival probability at time *t*, weighted by the inverse of the cumulative probability of censoring. The integrated Brier score (IBS) is then calculated as

$$IBS=\frac{1}{max(t_{i})}\int_{0}^{t_{i}}BS(t)dt$$

It can vary from 0 to 1. Two critical values for the (integrated) Brier score are 0.25 and 0.33, where 0.33 corresponds to predicting the risk by a uniformly distributed random number, and 0.25 corresponds to predicting a probability of 0.5 for every observation. This metric is evaluated at the end of the observation period in tables. As the models might be optimal in different periods in the follow-up time, the (unintegrated) Brier score, BS(*t*), will also be plotted as a function of time in prediction error curves [45]. In our study, the inverse probability of censoring weights are estimated independent of the values of the covariates, because censoring was exclusively caused by the maximum observation date in the data.

Time dependent AUC (AUC(*t*),[46, 89]). This is a generalization of the concordance statistic or *C*-index [47] so it can depend on time. The original *C*-index can be interpreted as the proportion of all pairs of subjects whose actual survival times are correctly ordered according to the estimated risk, among all pairs that actually can be compared (i.e., no pairs of censored/uncensored observations where the time of the censored observation is smaller). The AUC(*t*) differs from the C-index in that it only uses observations that are uncensored at time *t*. To correct for this, the inverse probability of censoring weights are applied to the uncensored observations, consisting of one divided by the Kaplan-Meier estimate of probability of censoring at time *t*. In this study, the AUC(*t*) metric is evaluated at each whole year.

Time dependent *R*^2^[49]. This is a proposed survival version of the R-squared criterion. It treats the Brier score (mean squared error) of the product-limit or Kaplan-Meier estimate as the reference model without covariates. It is calculated as

$$R^{2}(t)=1-\frac{BS(model|t)}{BS(KM|t)}$$

*R*^2^(*t*) is calculated at each year. Because the denominator is the same for all models on the same data, it will result in the same model selection when *BS*(*model*|*t*) would be used [44]. This metric is evaluated at every whole year of follow up time.

#### Linearizing transformations

In statistical practice, residual analysis is usually performed to detect nonlinearity. It is, however, hard to specify which transformation should be used based on these plots. Therefore, to account for nonlinearity in the linear statistical models, logistic regression analyses (binary case) and Cox regression analyses (censored data case) were fit using the generalized additive model formulation with respectively the logit link and the Cox link function [13] on the estimation data sets. Multiple nonlinear relations can then be effectively visualized by plotting the partial effect of these predictors, and these plots hint which transformation is most appropriate. We fit third degree P-splines with 4 degrees of freedom on the continuous predictors. We tried the natural log, inverse (1/*x*), quadratic and square root transform on visibly nonlinear terms and for each term chose the one that best approached linearity. The actual linearity of the resulting transformed term was then visually checked by modeling the P-spline version of the transformed predictors.

The data that were transformed to linear were input for all models that are inherently linear. For the binary case these were (*L*_1_- or *L*_2_-) logistic regression and (penalized) linear discriminant analysis. For the censored data these were (*L*_1_- and *L*_2_-) Cox-, split population Cox-, parametric, split population parametric, Cox boosting, partial least squares and Aalen models.

In previous research we attempted to include different interactions like gender × age, age × previous convictions, gender × previous convictions. Adding these parameters tended to deteriorate cross-validated performance, so we did not try them in this paper.

#### Model fitting procedure

In order to find the best tuning parameter settings for each algorithm, we used 10-fold cross validation (also known as rotation estimation) on the estimation part of the data.

For the binary case, the tuning parameter(s) yielding the highest accuracy was chosen. Because of the very low base rate in the sexual recidivism data, the Kappa statistic is used as the model selection criterion. For all survival analysis data, the integrated Brier score was chosen as the criterion for model/tuning parameter selection.

After the tuning/modeling stage is completed, the selected final models are fit on the complete estimation (training) sample and the final fit is established on the validation (test) sample.

Not all algorithms optimize the same loss function. Also, the optimized loss function is not always the same loss on which models are evaluated. For instance, Cox regression related techniques will optimize the semi-parametric Cox partial likelihood, the parametric survival analysis will optimize the full parametric likelihood while the integrated Brier score and *R*^2^ are used for evaluation. For optimal comparability, all algorithms would optimize the same loss function, and the loss function would be the evaluation criterion.

Some machine learning methods for binary outcome data tend to generate badly calibrated probabilities. Therefore, when needed Platt-calibrated probabilities [90] are generated.

## Results

The results are presented as tables in S2–S10 Tables. In the main text, only the graphical representations are included.

### Results binary outcome data

**Fig 1:**
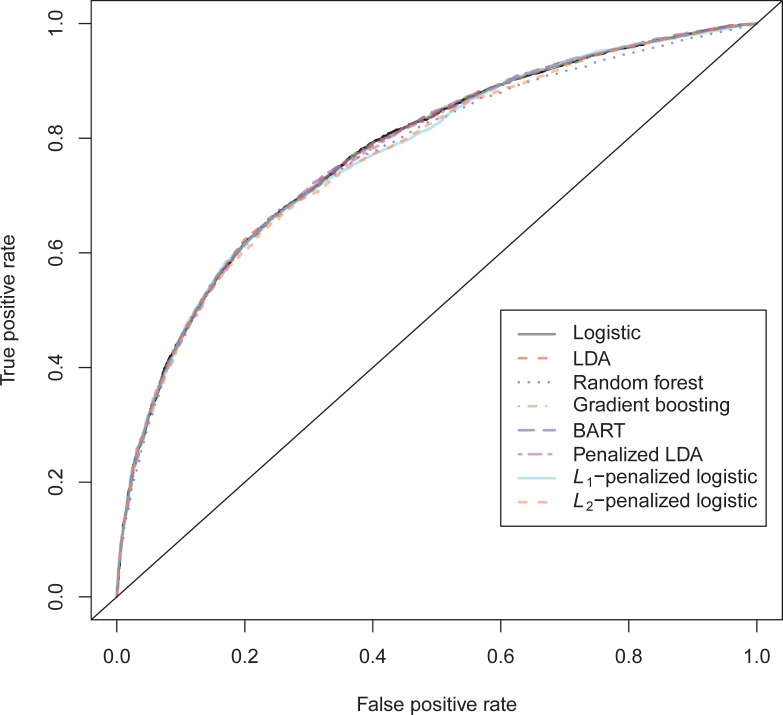
ROC-curves general recidivism test data.

**Fig 2:**
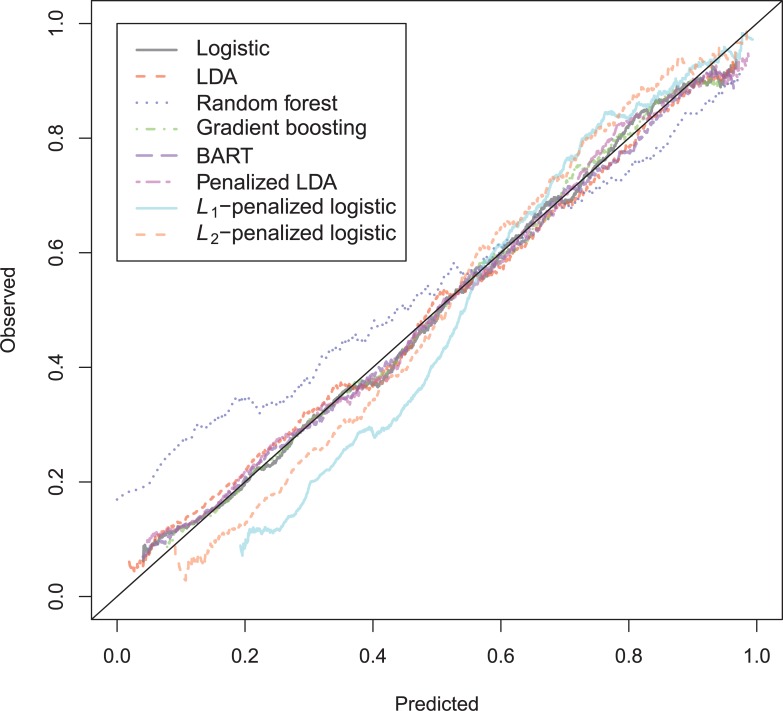
Calibration plots general recidivism test data.

**Fig 3:**
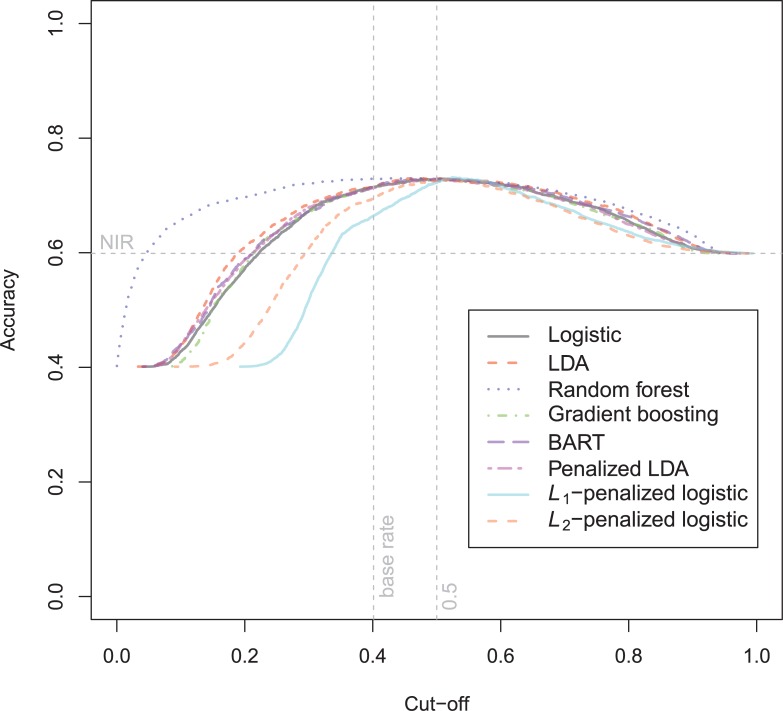
Accuracy plot general recidivism test data.

The general recidivism binary outcome data show that the different models are extremely similar in predictive performance (see S1 Table). There is hardly any variation in all criteria. Some salient values are the high accuracy of random forests when the cutoff is set at the base rate, and its relatively high calibration error. BART has a minimal improvement over logistic regression, as can be seen in the AUC, the calibration and the accuracy when sensitivity = specificity. Linear discriminant analysis has a negligibly larger value of the *H*-statistic. Differences of these magnitudes cannot be considered a substantial improvement and may disappear when using a different testing sample. Fig 1 reveals that all ROC-curves almost coincide. Only *L*_1_-, *L*_2_-penalized logistic regression and random forests are sometimes below the other lines, indicating decreased discriminative utility. The calibration plot in Fig 2 shows that random forests and *L*_1_- and *L*_*2*_-penalized logistic regression generate badly calibrated (pseudo-)probabilities. This property makes these models less useful for predicting the actual probabilities or for the formation of risk categories, but they may still remain relevant for classification and ordering tasks. The accuracy plot in Fig 3 shows that at the cutoff of .5, there is no variation between models in the accuracy. The effect of the penalty in *L*_1_- and *L*_2_-penalized logistic regression is that the probabilities become highly biased. Penalized LDA does however not seem to have this bias. An interesting property of the random forest average votes is that the accuracy does not vary much across cutoff points.

The results on the violent recidivism binary outcome data also show very little variation in performance (S2 Table). This time, stochastic gradient boosting (GBM) shows a minimal improvement on the *H*-statistic and the AUC, the RMSE and the accuracy at sensitivity = specificity. Linear discriminant analysis gives the highest accuracy at a cutoff of .5, whereas again the random forest has a substantially better accuracy at the base rate. Penalized discriminant analysis has the best AUC-value. BART achieves the best calibration error.

**Fig 4:**
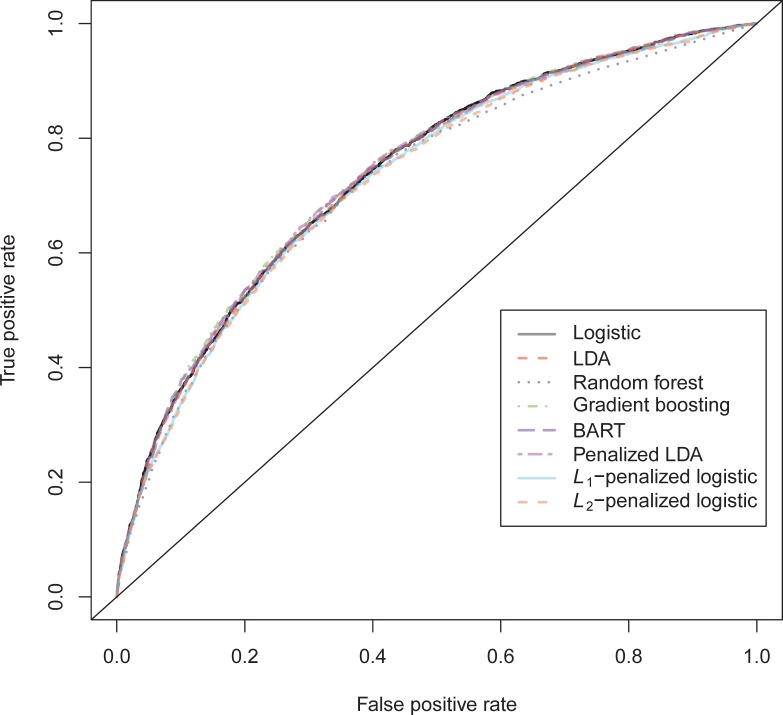
ROC-curves violent recidivism test data.

**Fig 5:**
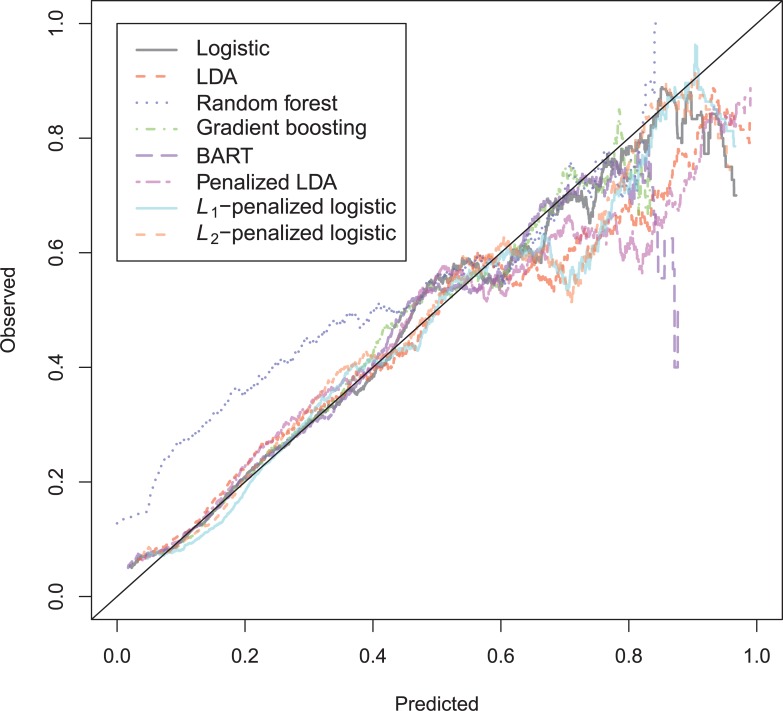
Calibration plots violent recidivism test data.

**Fig 6:**
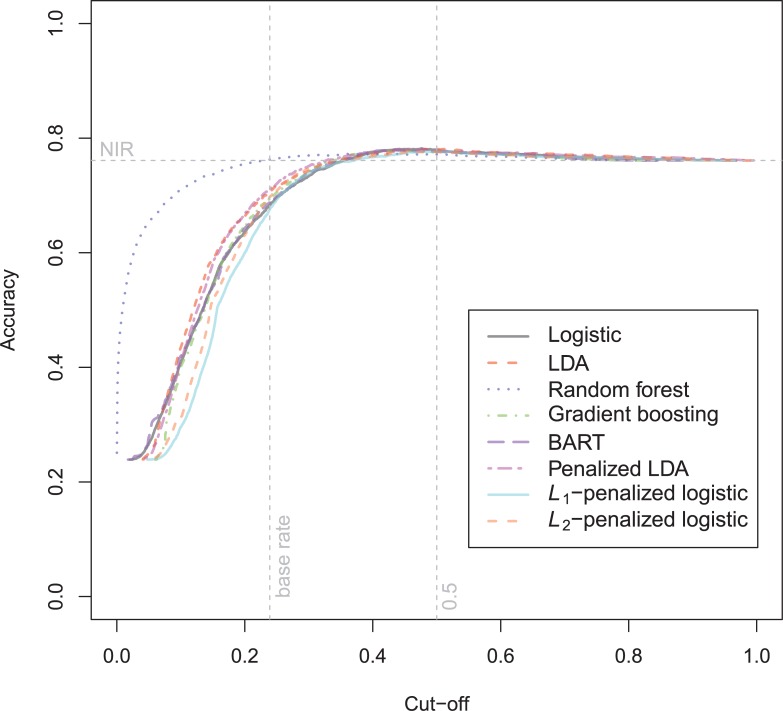
Accuracy plot violent recidivism test data.

The ROC-curves for the violent recidivism models in Fig 4 again show almost no variation. A small underperformance can be seen on random forests, *L*_1_- and *L*_2_-penalized logistic regression. In the calibration plots (Fig 5), the bad calibration of the random forest pseudo-probabilities is again evident. The other models do not seem to improve much over logistic regression on this criterion. The accuracy plot in Fig 6 shows that again at the 0.5 cutoff, there is hardly any variation in accuracy. In these data, *L*_1_- and *L*_2_-penalized logistic regression are not as biased as in the general recidivism data. This is most probably caused by the larger set of predictors, that increases variance in these models.

With the sexual recidivism data (S3 Table), *L*_1_-penalized logistic regression performs best by far in terms of H, AUC, RMSE and SAR, although the penalized discriminant analysis shows the best accuracy when sensitivity = specificity and calibration error, while LDA shows the best ACC(br). This suggests that regularization improves the generalization of the linear models on this data set, indicating a large amount of overfitting by logistic regression. Random forest also seems to overfit, as it performs only slightly better than logistic regression. LDA is much less prone to overfitting, than logistic regression as can be seen on the wide range of performance criteria, excepting RMSE, whereas GBM shows substantial improvement over simple logistic regression, as can be seen on most of the criteria, but it still performs much worse than *L*_1_-penalized logistic regression.

**Fig 7:**
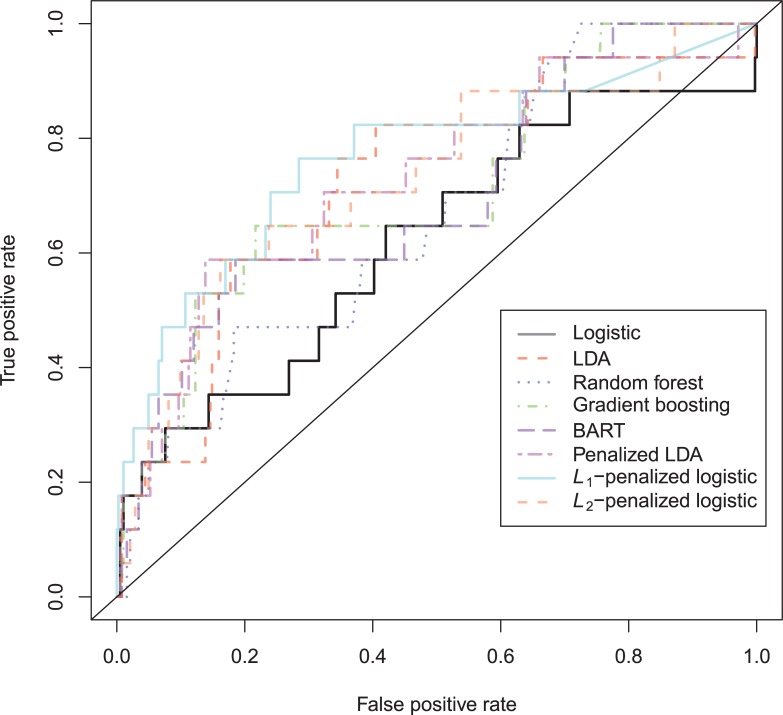
ROC-curves sexual recidivism test data.

**Fig 8:**
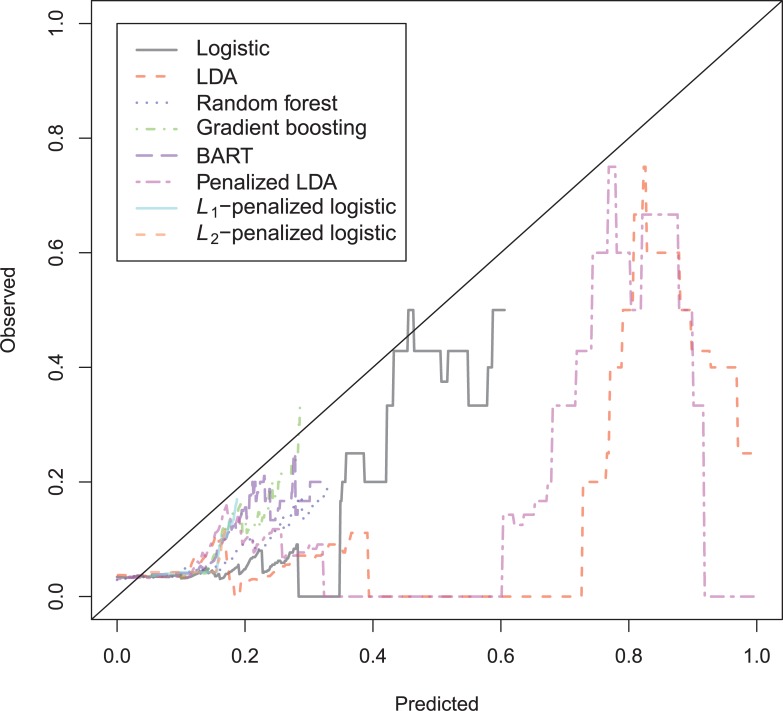
Calibration plots sexual recidivism test data.

**Fig 9:**
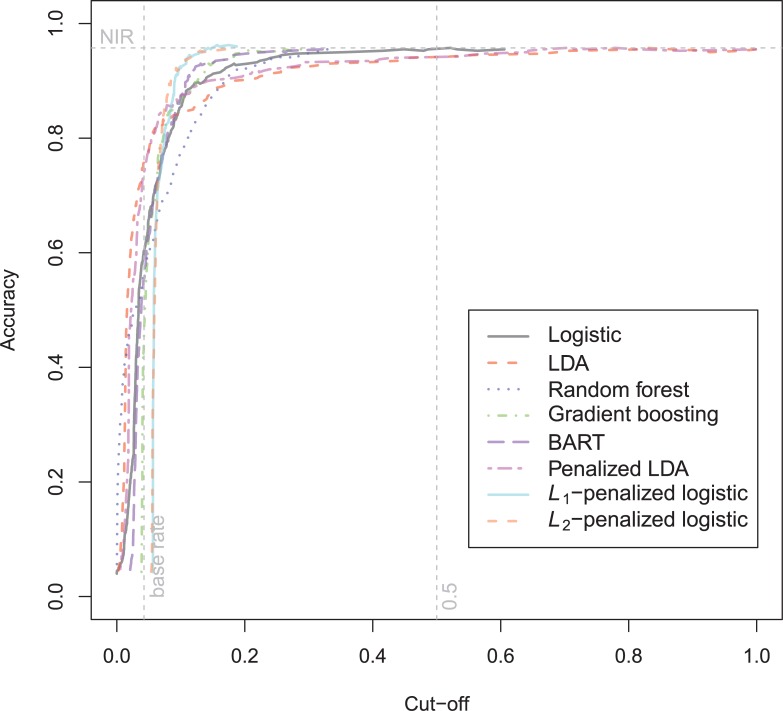
Accuracy plot sexual recidivism test data.

The plots in Fig 7 to 9 provide additional information. The ROC-curves (Fig 7) vary considerably, where *L*_1_-penalized logistic regression is highest curve up to a false positive rate of approximately 0.35. The lines of the different models cross beyond that point.

The calibration plot (Fig 8) is very hard to interpret, as most observations have a very small predicted probability. The penalized LDA seems to be able to stay closest to the ideal line of observed = predicted.

The Accuracy (Fig 9) plot shows that *L*_1_- and *L*_2_-penalized logistic regression obtain the highest accuracy. Only the line of *L*_1_-penalized logistic regression is able to reach above the no information rate of 0.958.

For the North Carolina prison data of 1978, the results (S4 Table) show that linear discriminant analysis has the best predictive performance, judging by the SAR. Its *H*-statistic is also the largest, although differences are very small. Again, the ACC at the base rate is highest in the random forest model. On the other hand, the best calibration error is attained by logistic regression. The best accuracy at the base rate and accuracy when specificity = sensitivity is attained by BART, which is one percent higher than logistic regression. *L*_1_-/*L*_2_-penalized logistic regression fail in these data. Again, the performance improvements seem only marginal.

**Fig 10:**
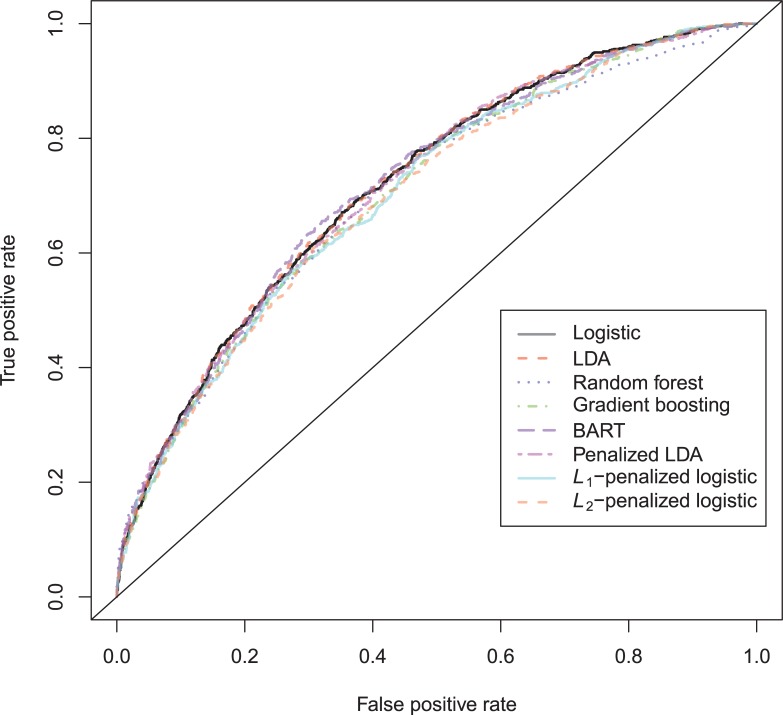
ROC-curves Schmidt & Witte 1978 test data.

The ROC-plot (Fig 10) shows that only Random Forest, gradient boosting, *L*_1_-/*L*_2_-penalized logistic models tend to underperform. The ROC-curves of the rest of the models more or less coincide.

**Fig 11:**
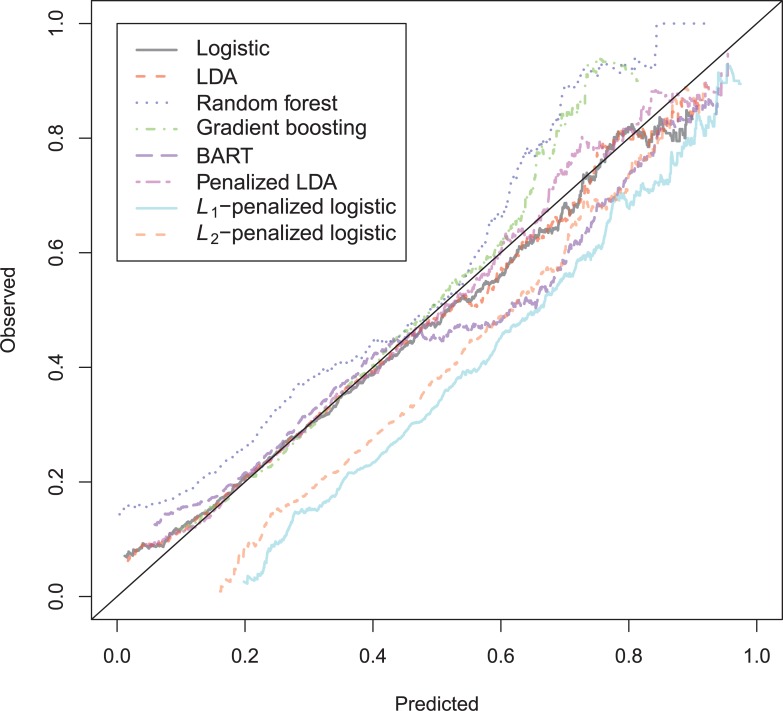
Calibration plot Schmidt & Witte 1978 test data.

The calibration plot (Fig 11) reveals very inaccurate probability estimates of *L*_1_-/*L*_2_-penalized logistic regression, and Random Forest on the complete range of probabilities, whereas the BART and gradient boosting are mostly badly calibrated in the upper region. The *L*_1_-/*L*_2_-models show severe overprediction which is caused by underfitting.

**Fig 12:**
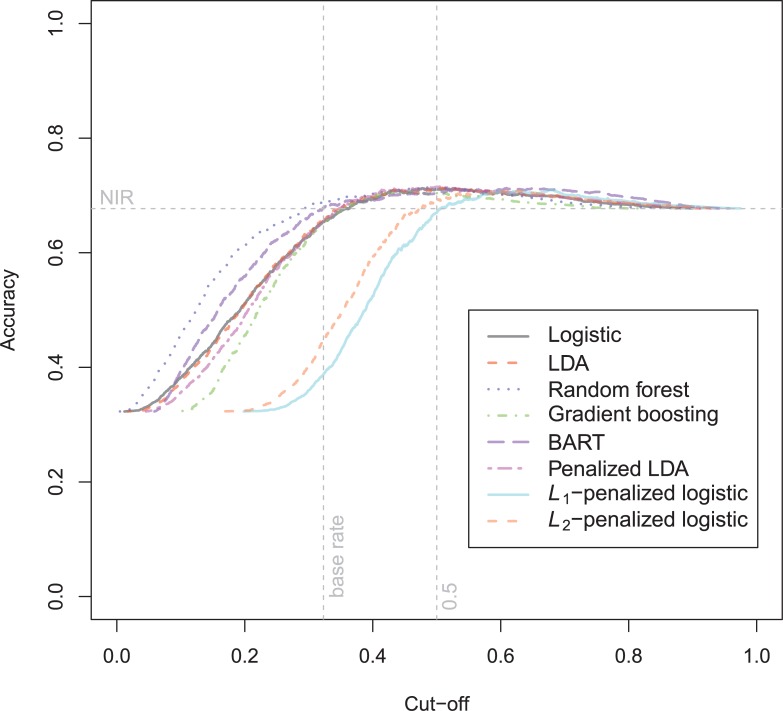
Accuracy plot Schmidt & Witte 1978 test data.

The accuracy plot (Fig 12) shows presence of severe overprediction by the *L*_1_-/*L*_2_-penalized models, as the entire accuracy curves are shifted to the right. By optimizing these models for accuracy, calibration is sacrificed.

**Fig 13:**
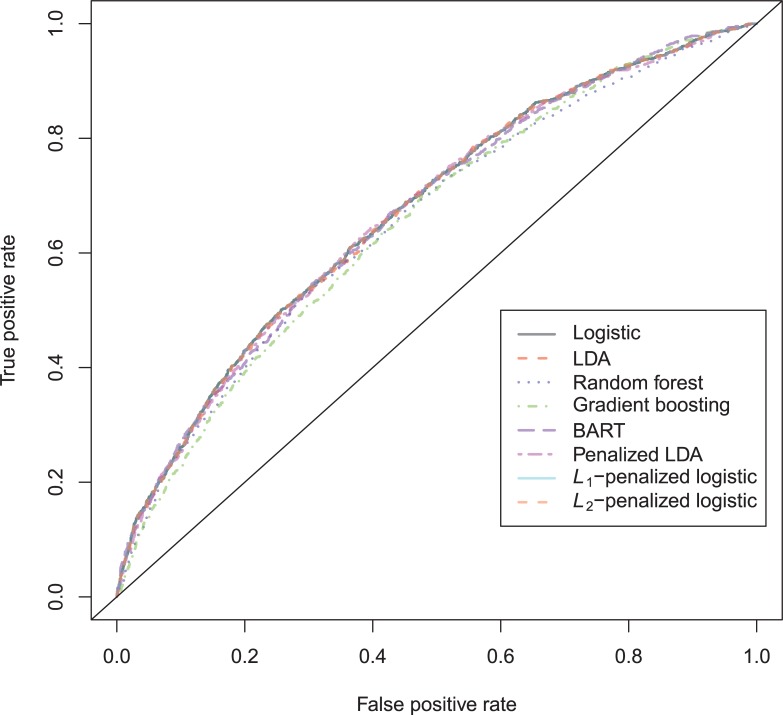
ROC-curves Schmidt & Witte 1980 test data.

The North Carolina prison data of 1980 is harder to predict than the 1978 data (see S5 Table). The maximum attained AUC is only 0.67. The best accuracy at the base rate is seen with the BART-model, but this model has worse calibrated probabilities in the upper region. Not a single model is able to improve upon the performance of the logistic regression, except on ACC(br) (random forest), and CAL (GBM). The area under the ROC-curve is notably smaller in the 1980 than in the 1978 data (Fig 13). The only models that show decreased discrimination are the stochastic gradient boosting, random forest and BART models.

**Fig 14:**
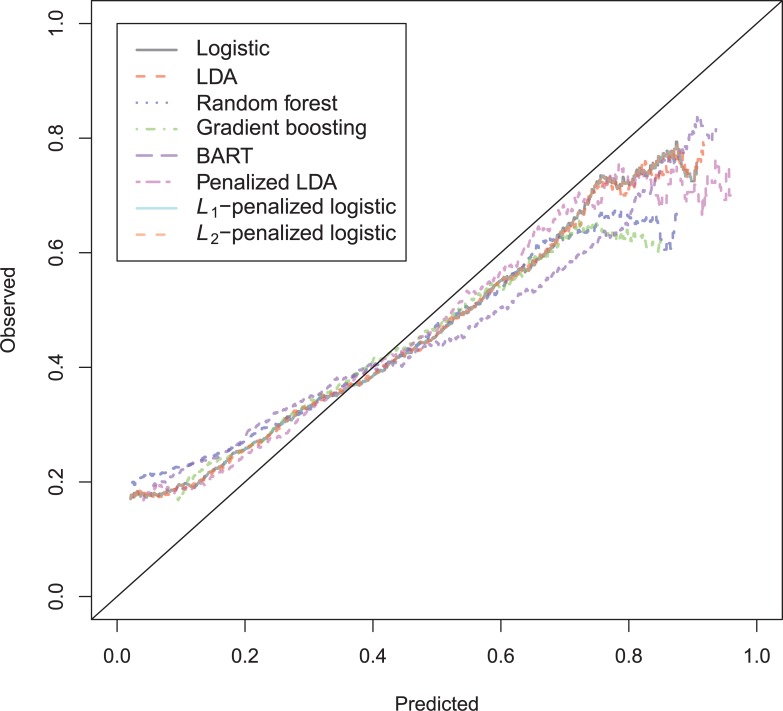
Calibration plot Schmidt & Witte 1980 test data.

The calibration plot (Fig 14) shows that it is hard for all models to obtain a reasonable calibration on this data set. Basically every model underpredicts in the lower probability region and overpredicts in the upper region. The calibration error is however largest in the flexible models, Random Forest, Gradient Boosting and BART. Again, the best calibration over the whole range of predicted probabilities is attained by the penalized LDA.

**Fig 15:**
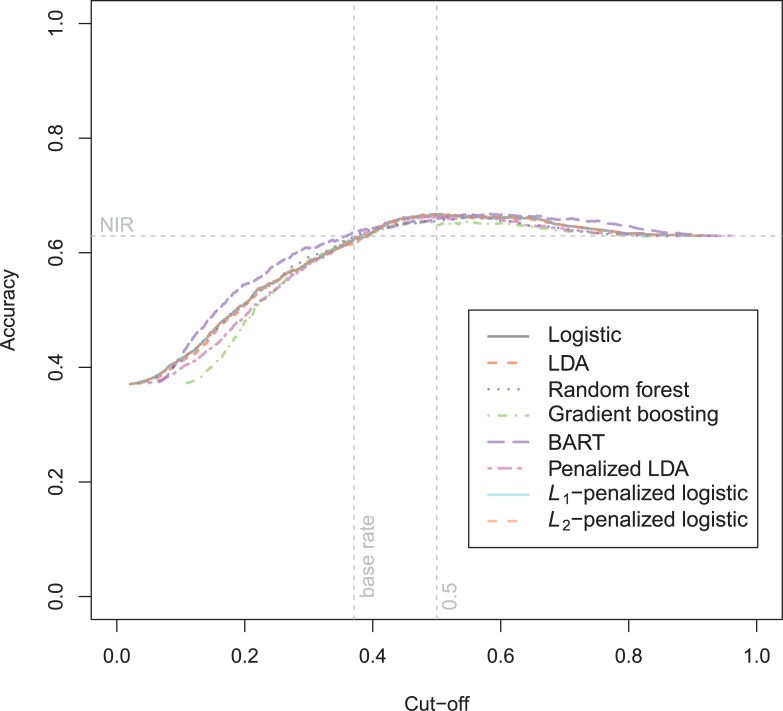
Accuracy plot Schmidt & Witte 1980 test data.

The accuracy curves are quite similar for the different models (Fig 15). BART tends to have a higher accuracy in the high and low areas of probability.

#### Summary across binary outcome data sets

There seems to be little room for improvement over logistic regression or LDA when data is appropriately linearized. An exception to the rule is that sometimes random forests or BART seem to do well on the accuracy when the base rate is used as a cutoff-score. However, this result is less relevant because this accuracy is never higher than the accuracy at cutoff = 0.5. Sometimes, *L*_1_-regularization seems to improve logistic regression.

### Results censored data

**Fig 16:**
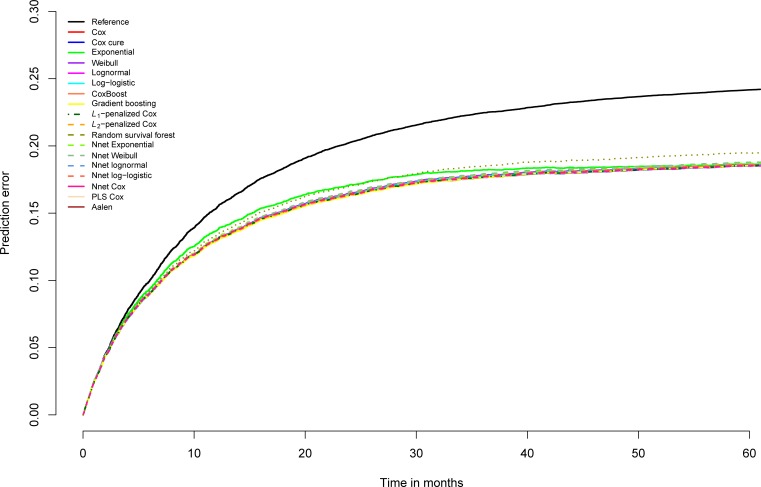
Brier scores general recidivism survival models over time, test data.

On general recidivism survival data, not much is gained in AUC by using other models than the standard survival model, the Cox regression model (S6 Table). On the AUC, the gradient boosting survival models show a tiny improvement for some years that is shared by the Cox cure model at 2 years. Random survival forest, split population models, neural network models, partial least squares and penalized Cox models show no improvement over Cox regression in these data. Contrary to the AUC, the R-squared statistics do however indicate improvements. Overall, there is virtually no distinction in the performance of the different models. The prediction error plot (Fig 16) graphically shows the similarity of the models. Only the exponential model and the random survival forest model depart negatively from the rest on prediction error. Notably, the difference between the prediction error of random survival forests and the other models increases with time, suggesting increasing instability.

**Fig 17:**
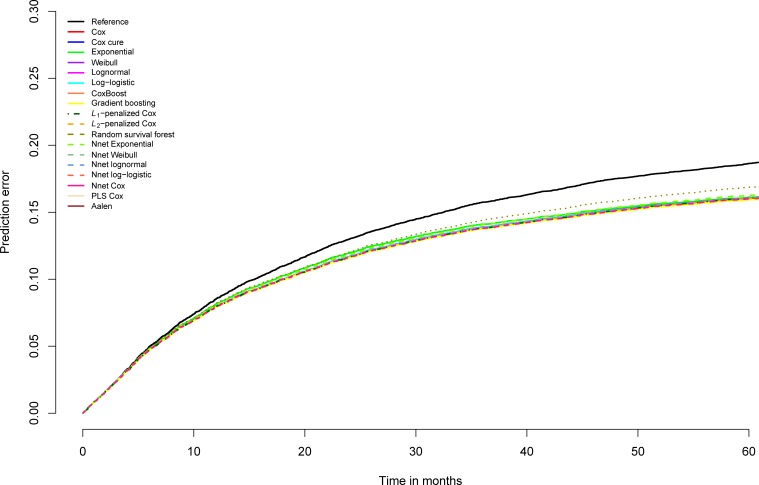
Brier scores violent recidivism survival models over time, test data.

The results on the violent recidivism data (S7 Table) show that gradient boosting Cox model achieves the largest improvement upon the standard Cox model, but the improvement is very small. The rest performs in a more or less comparable way, with the random survival forest as the negative exception. This model is poor at discrimination (AUC), prediction error (IBS), and explained variance. The prediction error plot (Fig 17) shows almost no variation in the prediction error curves. Only the random survival forest, exponential, and the neural network exponential models seem to perform slightly worse. As in the general recidivism data, the random survival forest prediction error increases relative to the other models, the longer the observation time.

**Fig 18:**
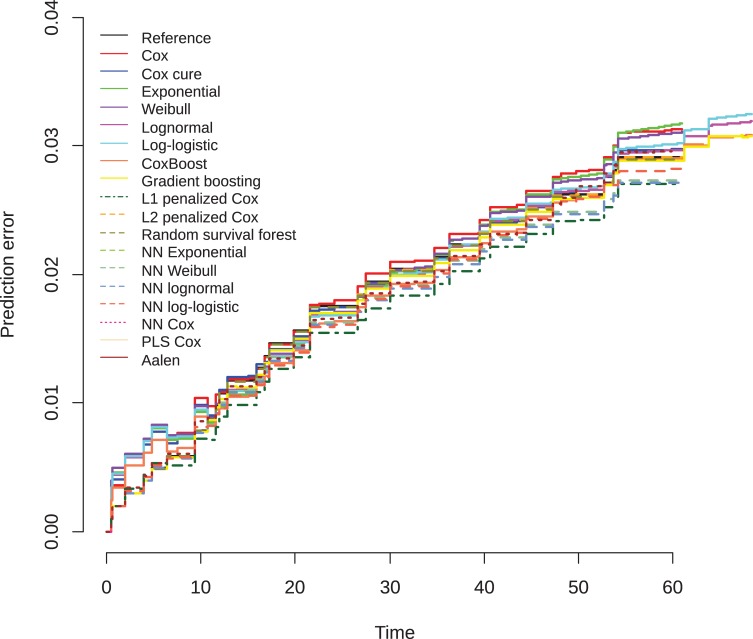
Brier scores sexual recidivism survival models over time, test data.

As in the binary outcome data results on sexual recidivism again show more differentiation (S8 Table), but mostly models do worse than the standard Cox model. The log-logistic model performs slightly better on the AUC on years 2–5, whereas the Weibull model has the highest AUC in one year after the index case. The best IBS and *R*^2^-values are however attained by the *L*_1_-penalized Cox regression, but at the expense of worse AUC’s. Fig 18 shows that the best prediction error over the complete time range is attained by *L*_1_-penalized Cox regression and neural network Cox. Both methods control overfitting by shrinking the coefficients towards zero. Apparently in survival analysis, as in the binary case of sexual recidivism, the data are quickly overfit when using the complete set of predictors.

**Fig 19:**
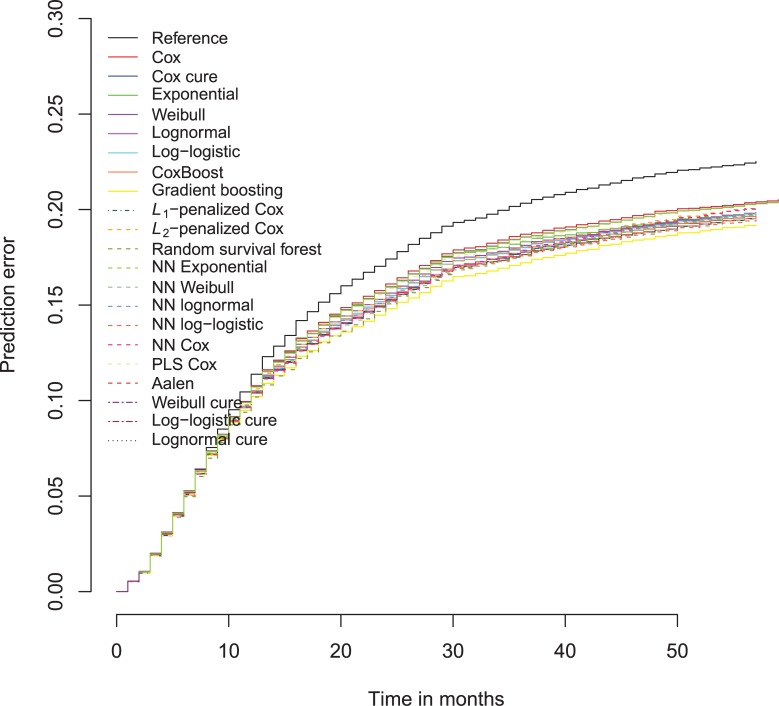
Brier scores survival models Schmidt Witte 1978 data over time, test data.

Stochastic gradient boosting survival trees clearly outperform all other models on the North Carolina prison data of 1978. The performance of the rest of the models is substantially lower on all performance criteria (S9 Table). This is also evident from the prediction error curves (Fig 19). The model especially performs better on the longer observation times.

**Fig 20:**
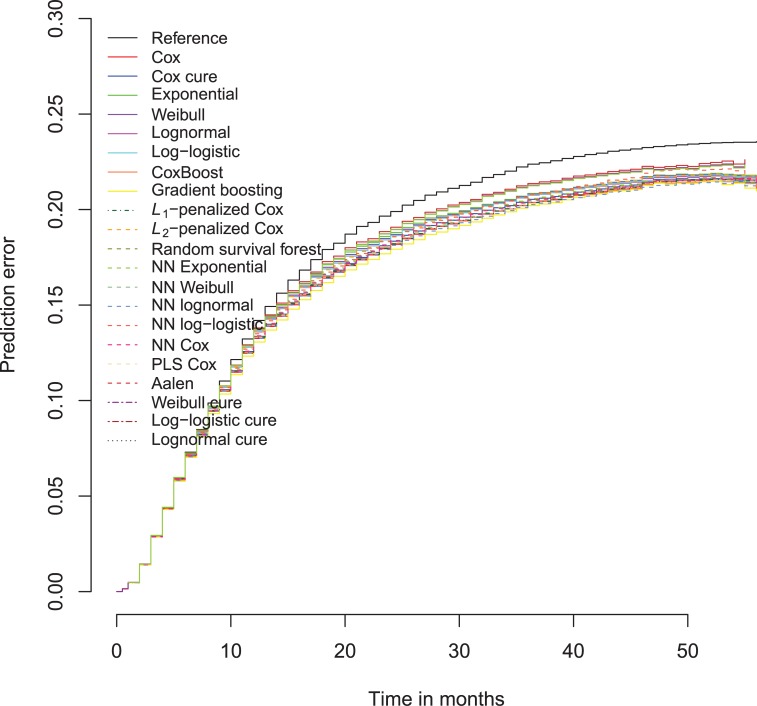
Brier scores survival models Schmidt Witte 1978 data over time, test data.

The North Carolina prison data of 1980 is also best predicted by stochastic gradient boosting survival trees, but the differences are less convincing than in the 1978 data (Fig 20 and S10 Table). Moreover, the neural network lognormal models have a slightly better *R*^2^ at one and four years after the index case.

#### Summary across censored outcome datasets

Often, Cox regression is not improved upon in our selection of datasets. However, in the North Carolina prison data, stochastic gradient boosting seems to pick up extra information for prediction. In each data set, the prediction of random survival forests are very unstable, especially on the longer observation times. In the dataset with the least number of recidivists, the sexual recidivism data, *L*_1_-Cox provides better prediction than standard Cox.

## Discussion

### Statistics vs machine learning

In past years, concerns were raised whether using classical statistical modeling in risk prediction was an optimal strategy. Especially random forests and stochastic gradient boosting were seen as promising new techniques that exploit subtle, complex interaction effects and nonlinearity. In this paper, we investigated in five data sets whether random forests, stochastic gradient boosting, Bayesian regression trees (BART) and regularized logistic regression and regularized LDA improve predictions over standard logistic regression and LDA. An important condition for this study was that for inherently linear models, model specification involved transforming continuous variables to accommodate for nonlinearity. Nonlinear continuous predictors were identified by visually exploring generalized additive models and subsequently transforming these predictors manually to linearity using a handful of canonical transformations. For all five data sets with a binary four year recidivism outcome, overall no substantial improvement of the new methods was found over the traditional statistical modeling approaches, neither in the correct ordering of risk scores, calibration of the predicted probabilities or classification accuracy. The only exception was that *L*_1_-logistic regression could find substantially higher classification accuracy in the highly unbalanced sexual recidivism data. In this data, overfitting by allowing too many parameters was prevented. Here regularization seems to give more gains in predictive accuracy than intricate nonlinearity and/or interactions, although it reduces calibration due to biasing the coefficients. Random forests are unable to generate well-calibrated pseudo-probabilities, even after using Platt scaling. Their application seems to be limited to risk score ordering or classification.

In this paper we also studied whether the prediction of survival analysis models can be improved by using generalizations of machine learning techniques for censored data. In this type of data, stochastic gradient boosting models did establish a substantial improvement in prediction error and time dependent AUC over the classical Cox model in the smaller data sets. This result indicates that classical survival modeling and possibly machine learning survival modeling too, leave room for improvement. Overall, random survival forest models did notably worse than standard statistical modeling in censored data. Specifically, the relative performance discrepancy exacerbates as the observation time increases.

In sum, on the binary recidivism data sets that we studied, a manually specified traditional model performs as well as flexible automatic machine learning models. In this case, one might therefore choose either strategy for finding a good model. There are however several reasons to prefer a traditional model over a more flexible machine learning model, and therefore putting some effort into finding linearizing transformations. First, having few parameters makes models less likely to overfit the data (i.e. a low variance model), which is especially advantageous in smaller samples. Second, transparency is lost by using a machine learning model instead of a statistical model. Because of the opacity of machine learning models, it is less clear how the predictors are related to the outcome. Because of that, the analyst is less likely to detect suspicious counter-intuitive results. Moreover, it is harder to foresee how the model will extrapolate to data values or combinations of data values not seen in the development sample. On the other hand, classical statistical models can transparently be assessed for strange results using common model diagnostics and assessment of the model coefficients and standard errors. Finally, in the case of individual prediction, uniqueness of probability are lost when using stochastic techniques like stochastic gradient boosting and flexible models that depend on random starting configurations like neural networks. These models will yield non-unique individual probabilities and classifications, introducing an element of arbitrariness in individual decision making or risk categorization.

Another issue, related to transparency is interpretability or comprehensibility (see [91] for an overview of interpretability in classification models), which is important in the development of decision instruments or risk category formation and not so much in other prediction scenarios. When using a black-box algorithm, it is simply not understandable how it arrived at the prediction. The practitioner will find it hard to trust the output of the model, and it becomes harder to ‘sell’ to the practitioners who must apply the resulting instrument. However, the interpretability of a traditional statistical model should not be overstated, as it is not trivial to explain how logistic regression with nonlinear terms arrives at a prediction to a layman.

A possible strategy to assess whether a well predicting but still interpretable model can be specified might be as follows. A classical low parametric statistical model should be specified by first exploring non-linearity and including transformed predictors. Then, an adaptive tree-based boosting procedure like adaBoost, stochastic gradient boosting or Bayesian additive regression trees could be fit to detect possible model misspecification. If the machine learning does not indicate possible improvement, the classical low-parametric model can be used.

In this study and in previous comparison studies in the criminological literature, data sets with a relatively small number of variables were used. It is to be expected that when datasets with hundreds of continuous variables are used, it will become impracticable to manually transform many continuous predictors. In that circumstance, more flexible techniques from machine learning might offer a more convenient solution for building a nonlinear prediction model.

### Manual versus automatic tuning

Tuning machine learning methods requires the researcher to test out different configurations of tuning parameters, which can hardly be exhaustive. A shortcoming of the current study might be that some optimal values of tuning parameters have been missed, especially in the case of multiple real valued tuning parameters. To alleviate this problem, some have proposed meta-methods for optimizing the tuning parameters automatically, e.g. [92]. They propose to use their intricate sequential model-based algorithm configuration (SMAC) for the problem of combined algorithm and hyperparameter selection (CASH). This algorithm keeps track of all configurations of tuning parameters and the associated accuracy and searches promising directions in the tuning parameter space. It is still however not guaranteed to find a better model than manual tuning or even an equally good model. The algorithm is implemented in an automated version of the open source Weka machine learning software suite[93], Auto-Weka[94]. A limitation of the software is that it is limited to binary or continuous outcomes, accuracy optimization and is not suitable survival data. A 72 hour run of Auto-Weka 0.5 on every data set in this paper of using the default settings that also involved testing different algorithms for automatic variable selection, did *not* however find any improvement upon the final models found. On all three Dutch data sets, logistic regression with a small *L*_2_-penalty was automatically selected as the best model. The Caroline prison data were automatically fit using a bagging of decision trees (the 1978 data) and a neural network (the 1980 data).

### Meta-models

As in this study, when searching for the best prediction model, researchers often end up in the situation that very different models tend to provide the same predictive accuracy (the ‘Rashomon effect’ [10]). A popular approach to obtain a better prediction model is to combine the predictions of different models into one predictor by using a meta-model. This strategy is however only useful when predicted probabilities from different models differ substantially for individuals. In this case, different strong models may emphasize different aspects of the data and they might be combined into one predictor [95]. Already in 1992, [96] suggested using the out-of-sample predicted probabilities of the *k*-fold estimated models of the learning data for as input variables for a single meta-classifier, in the context of neural network modeling, called stacked generalization (also known as stacking or blending). The resulting classifier asymptotically performs at least as good as the best of the separate classifiers. Thus, stacking is a way to combine the strengths and average out the weaknesses, in other words to use the wisdom of a crowd of heterogeneous *strong* learners. Algorithms that win in competitions like those of Kaggle are almost always ensembles of models (see e.g.[97]). The actual gains in predictive performance are however often very small. Nevertheless, even small improvements of predictive accuracy in a decision context could result into a difference of hundreds of crimes [24]. The stacking approach has recently been generalized to the survival analysis domain[98].

Combining strong classification models into one classifier does come at a cost. It makes the final ‘model’ even less transparent than its constituent parts. Moreover, the amount of possible combinations of models and meta-learners is practically unlimited and finding ‘the best’ model is a difficult task. As long as there is no deep theoretical understanding of why (meta-)models perform well in certain situations, it will be a question of trial and error and exchange of purely experiential knowledge that keeps the practice of applying predictive models getting better instead of directed improvement by scientific insight.

## Supporting information

S1 File Used R-packages. (DOCX)

S2 File Zipfile of R, Python and SPSS Source code. (ZIP)

S1 Table Predictive performance general recidivism (4 year reconviction yes/no). (DOCX)

S2 Table Predictive performance violent recidivism (4 year reconviction yes/no). (DOCX)

S3 Table Predictive performance sexual recidivism (4 year reconviction yes/no). (DOCX)

S4 Table Predictive performance Schmidt and Witte 1978 data (4 year reconviction yes/no). (DOCX)

S5 Table Predictive performance Schmidt and Witte 1980 data (4 year reconviction yes/no). (DOCX)

S6 Table Predictive performance general recidivism (survival data). (DOCX)

S7 Table Predictive performance violent recidivism (survival data). (DOCX)

S8 Table Predictive performance sexual recidivism (survival data). (DOCX)

S9 Table Predictive performance Schmidt and Witte 1978 data (survival data). (DOCX)

S10 Table Predictive performance Schmidt and Witte 1980 data (survival data). (DOCX)
