# Supplementary material for: Optimizing predictive performance of criminal recidivism models using registration data with binary and survival outcomes
Source: PLoS One. 2019 Mar 8;14(3):e0213245. doi: 10.1371/journal.pone.0213245 (PMC6407787; doi:10.1371/journal.pone.0213245)
Supplement: S1 File — (DOCX) [file pone.0213245.s001.docx]

**S1 used R-packages**

The following R packages were used for modeling:

- BayesTree v0.3-1.1
- CoxBoost v1.4
- Penalized v0.9-42
- caret v5.16-24
- doSNOW v1.0.12
- foreach v1.4.1
- gam v1.08
- gbm v2.1
- a modified version of the library gfcure (WinR)
- iterators v1.0.6
- nnet v7.3-6
- pec v2.2.9
- plsRcox v0.9
- randomForestSRC v1.1.0
- rms v3.6-3
- smcure v2.0
- snow v0.3-12
- survnnet v1.1-3
- timereg v1.7.7
