# Supplementary material for: Optimizing predictive performance of criminal recidivism models using registration data with binary and survival outcomes
Source: PLoS One. 2019 Mar 8;14(3):e0213245. doi: 10.1371/journal.pone.0213245 (PMC6407787; doi:10.1371/journal.pone.0213245)
Supplement: S1 Table — (DOCX) [file pone.0213245.s003.docx]

**S1 Table. Predictive performance general recidivism (4 year reconviction yes/no).**

|  | *H* | AUC | ACC | ACC(br) | RMSE | SAR | SAR(br) | CAL | ACC(SPEC=SENS) |
| --- | --- | --- | --- | --- | --- | --- | --- | --- | --- |
| Logistic regression | 0.268 | 0.776 | 0.728 | 0.714 | 0.430 | 0.692 | 0.687 | 0.034 | 0.703 |
| LDA | **0.269** | 0.776 | 0.729 | 0.716 | 0.430 | 0.692 | 0.688 | 0.036 | 0.703 |
| Random forest^*^ | 0.260 | 0.768 | 0.729 | **0.724** | 0.433 | 0.688 | 0.686 | 0.043 | 0.703 |
| GBM | 0.267 | 0.776 | 0.727 | 0.714 | 0.430 | 0.691 | 0.686 | 0.035 | 0.704 |
| BART | 0.269 | **0.778** | 0.729 | 0.714 | 0.429 | 0.692 | 0.687 | 0.028 | **0.707** |
| PDA | 0.269 | 0.776 | 0.729 | 0.716 | 0.430 | 0.692 | **0.688** | 0.035 | 0.700 |
| *L*_1_-logistic regression | 0.184 | 0.731 | **0.776** | 0.676 | **0.399** | **0.703** | 0.669 | 0.039 | 0.669 |
| *L*_2_-logistic regression | 0.260 | 0.769 | 0.725 | 0.702 | 0.434 | 0.687 | 0.679 | **0.054** | 0.700 |

*The Platt calibrated version of this model performed slightly better.
