# Supplementary material for: Optimizing predictive performance of criminal recidivism models using registration data with binary and survival outcomes
Source: PLoS One. 2019 Mar 8;14(3):e0213245. doi: 10.1371/journal.pone.0213245 (PMC6407787; doi:10.1371/journal.pone.0213245)
Supplement: S2 Table — (DOCX) [file pone.0213245.s004.docx]

**S2 Table. Predictive performance violent recidivism (4 year reconviction yes/no).**

|  | *H* | AUC | ACC | ACC(br) | RMSE | SAR | SAR(br) | CAL | ACC(SPEC=SENS) |
| --- | --- | --- | --- | --- | --- | --- | --- | --- | --- |
| Logistic regression | 0.198 | 0.739 | 0.779 | 0.683 | 0.395 | 0.708 | 0.676 | 0.034 | 0.672 |
| LDA | 0.197 | 0.739 | **0.781** | 0.706 | 0.397 | 0.708 | 0.683 | 0.038 | 0.675 |
| Random forest^*^ | 0.179 | 0.724 | 0.772 | **0.734** | 0.402 | 0.698 | 0.685 | 0.056 | 0.668 |
| GBM | **0.201** | 0.741 | 0.777 | 0.670 | **0.395** | 0.707 | 0.672 | 0.036 | **0.680** |
| BART | 0.199 | 0.740 | 0.776 | 0.687 | 0.395 | 0.707 | 0.677 | **0.031** | 0.677 |
| PDA | 0.200 | **0.742** | 0.779 | 0.713 | 0.396 | **0.708** | **0.686** | 0.038 | 0.679 |
| *L*_1_-logistic regression | 0.184 | 0.731 | 0.776 | 0.676 | **0.399** | **0.703** | 0.669 | 0.039 | 0.669 |
| *L*_2_-logistic regression | 0.180 | 0.728 | 0.776 | 0.697 | 0.399 | 0.702 | 0.675 | 0.034 | 0.670 |

*The Platt calibrated version of this model performed slightly better.
