# Supplementary material for: Optimizing predictive performance of criminal recidivism models using registration data with binary and survival outcomes
Source: PLoS One. 2019 Mar 8;14(3):e0213245. doi: 10.1371/journal.pone.0213245 (PMC6407787; doi:10.1371/journal.pone.0213245)
Supplement: S3 Table — (DOCX) [file pone.0213245.s005.docx]

**S3 Table. Predictive performance sexual recidivism (4 year reconviction yes/no)**

|  | H | AUC | ACC | ACC(br) | RMSE | SAR | SAR(br) | CAL | ACC(SPEC=SENS) |
| --- | --- | --- | --- | --- | --- | --- | --- | --- | --- |
| Logistic regression | 0.155 | 0.620 | **0.958** | 0.605 | 0.203 | 0.791 | 0.674 | 0.016 | 0.588 |
| LDA | 0.256 | 0.724 | 0.943 | **0.760** | 0.226 | 0.814 | **0.753** | 0.015 | 0.658 |
| Random forest | 0.166 | 0.664 | **0.958** | 0.570 | 0.205 | 0.805 | 0.676 | 0.035 | 0.589 |
| GBM | 0.273 | 0.710 | **0.958** | 0.458 | 0.198 | 0.823 | 0.656 | 0.023 | 0.647 |
| BART | 0.178 | 0.720 | 0.710 | 0.662 | 0.435 | 0.665 | 0.649 | 0.036 | 0.664 |
| PDA | 0.234 | 0.747 | 0.945 | 0.693 | 0.214 | 0.826 | 0.742 | **0.014** | 0.705 |
| *L*_1_-logistic regression^*^ | **0.324** | **0.765** | **0.958** | 0.043 | **0.192** | **0.843** | 0.538 | 0.028 | **0.708** |
| *L*_2_-logistic regression | 0.254 | 0.728 | **0.958** | 0.043 | 0.201 | 0.828 | 0.523 | 0.032 | 0.647 |

*The Platt calibrated version of this model performed better.
