# Supplementary material for: Optimizing predictive performance of criminal recidivism models using registration data with binary and survival outcomes
Source: PLoS One. 2019 Mar 8;14(3):e0213245. doi: 10.1371/journal.pone.0213245 (PMC6407787; doi:10.1371/journal.pone.0213245)
Supplement: S4 Table — (DOCX) [file pone.0213245.s006.docx]

**S4 Table. Predictive performance Schmidt and Witte 1978 data (4 year reconviction yes/no)**

|  | H | AUC | ACC | ACC(br) | RMSE | SAR | SAR(br) | CAL | ACC(SPEC=SENS) |
| --- | --- | --- | --- | --- | --- | --- | --- | --- | --- |
| Logistic regression | 0.173 | 0.719 | 0.711 | 0.655 | 0.435 | 0.665 | 0.646 | **0.030** | 0.657 |
| LDA | 0.175 | **0.720** | **0.713** | 0.654 | 0.435 | **0.666** | 0.647 | 0.032 | 0.654 |
| Random forest | 0.160 | 0.702 | 0.711 | **0.687** | 0.444 | 0.656 | 0.649 | 0.072 | 0.053 |
| GBM^*^ | 0.157 | 0.705 | 0.704 | 0.652 | 0.441 | 0.656 | 0.639 | 0.045 | 0.643 |
| BART | **0.178** | 0.719 | 0.709 | 0.663 | **0.435** | 0.664 | **0.649** | 0.038 | **0.667** |
| PDA | 0.174 | 0.717 | **0.713** | 0.657 | 0.435 | 0.665 | 0.646 | 0.034 | 0.651 |
| *L*_1_-logistic regression | 0.159 | 0.704 | 0.670 | 0.388 | 0.467 | 0.636 | 0.541 | 0.157 | 0.641 |
| *L*_2_-logistic regression | 0.151 | 0.700 | 0.686 | 0.470 | 0.456 | 0.643 | 0.571 | 0.119 | 0.642 |

*Because R crashed when fitting gradient boosting, we fitted this model using the scikit learn library (Pedregosa et al., 2005) in Python.
