# Supplementary material for: Optimizing predictive performance of criminal recidivism models using registration data with binary and survival outcomes
Source: PLoS One. 2019 Mar 8;14(3):e0213245. doi: 10.1371/journal.pone.0213245 (PMC6407787; doi:10.1371/journal.pone.0213245)
Supplement: S5 Table — (DOCX) [file pone.0213245.s007.docx]

**S5 Table. Predictive performance Schmidt and Witte 1980 data (4 year reconviction yes/no)**

|  | H | AUC | ACC | ACC(br) | RMSE | SAR | SAR(br) | CAL | ACC(SPEC=SENS) |
| --- | --- | --- | --- | --- | --- | --- | --- | --- | --- |
| Logistic regression | **0.117** | **0.672** | **0.667** | 0.623 | **0.463** | **0.625** | **0.611** | 0.052 | **0.620** |
| LDA | 0.115 | 0.671 | 0.664 | 0.616 | 0.463 | 0.624 | 0.608 | 0.050 | 0.615 |
| Random forest | 0.095 | 0.656 | 0.658 | **0.628** | 0.469 | 0.615 | 0.605 | 0.059 | 0.613 |
| GBM^*^ | 0.092 | 0.654 | 0.651 | 0.622 | 0.468 | 0.612 | 0.602 | **0.047** | 0.608 |
| BART | 0.113 | 0.669 | 0.666 | 0.625 | 0.463 | 0.624 | 0.611 | 0.050 | 0.617 |
| PDA | 0.093 | 0.654 | 0.499 | 0.397 | 0.528 | 0.542 | 0.508 | 0.249 | 0.609 |
| *L*_1_-logistic regression | 0.093 | 0.654 | 0.499 | 0.397 | 0.528 | 0.542 | 0.508 | 0.249 | 0.609 |
| *L*_2_-logistic regression | 0.093 | 0.654 | 0.507 | 0.401 | 0.521 | 0.547 | 0.511 | 0.234 | 0.606 |

*Because R crashed when fitting gradient boosting, we fitted this model using the scikit learn library (Pedregosa et al., 2005) in Python.
