# Supplementary material for: Optimizing predictive performance of criminal recidivism models using registration data with binary and survival outcomes
Source: PLoS One. 2019 Mar 8;14(3):e0213245. doi: 10.1371/journal.pone.0213245 (PMC6407787; doi:10.1371/journal.pone.0213245)
Supplement: S6 Table — (DOCX) [file pone.0213245.s008.docx]

**S6 Table. Predictive performance general recidivism (survival data)**

|  | AUC  (1 yr) | AUC (2yrs) | AUC  (3 yrs) | AUC  (4 yrs) | AUC  (5 yrs) | IBS  (4 yrs) | R^2^  (1 yr) | R^2^  (2 yrs) | R^2^  (3 yrs) | R^2^  (4 yrs) | R^2^  (5 yrs) |
| --- | --- | --- | --- | --- | --- | --- | --- | --- | --- | --- | --- |
| Cox | 73.8 | 73.2 | 72.8 | 72.8 | 72.6 | **0.154** | 0.154 | 0.191 | 0.212 | 0.229 | 0.234 |
| Cox cure | 73.8 | **73.3** | 72.9 | 72.8 | 72.6 | 0.159 | 0.158 | 0.194 | **0.214** | 0.229 | 0.234 |
| Exponential | 73.8 | 73.2 | 72.8 | 72.8 | 72.6 | 0.155 | 0.109 | 0.156 | 0.189 | 0.216 | 0.228 |
| Weibull | 73.8 | 73.2 | 72.8 | 72.8 | 72.6 | **0.154** | 0.151 | 0.185 | 0.206 | 0.224 | 0.232 |
| Lognormal | 73.8 | 73.2 | 72.9 | 72.8 | 72.6 | **0.154** | 0.156 | 0.191 | 0.210 | 0.225 | 0.231 |
| Loglogistic | 73.8 | 73.2 | 72.9 | 72.8 | 72.6 | 0.155 | 0.154 | 0.189 | 0.209 | 0.226 | 0.233 |
| Cox boosting | 73.6 | 73.0 | 72.6 | 72.5 | 72.4 | **0.154** | 0.153 | 0.190 | 0.210 | 0.225 | 0.230 |
| Gradient boosting | **74.0** | **73.3** | **73.0** | **72.9** | **72.7** | **0.154** | **0.160** | **0.195** | 0.214 | 0.228 | 0.233 |
| *L*_1_-Cox | 73.8 | 73.2 | 72.8 | 72.8 | 72.6 | **0.154** | 0.155 | 0.192 | 0.212 | 0.228 | 0.233 |
| *L*_2_-Cox | 73.8 | 73.2 | 72.8 | 72.8 | 72.6 | **0.154** | 0.155 | 0.192 | 0.213 | 0.228 | 0.233 |
| Random survival forest | 73.8 | 73.2 | 72.9 | 72.8 | 72.6 | 0.155 | 0.151 | 0.188 | 0.210 | 0.225 | 0.231 |
| Neural network (exponential) | 73.7 | 73.1 | 72.8 | 72.8 | 72.6 | **0.154** | 0.154 | 0.192 | 0.213 | **0.230** | **0.236** |
| Neural network (Weibull) | 73.7 | 73.1 | 72.6 | 72.6 | 72.4 | 0.162 | 0.116 | 0.151 | 0.168 | 0.177 | 0.181 |
| Neural network (lognormal) | 73.1 | 72.5 | 72.2 | 72.2 | 72.0 | 0.157 | 0.144 | 0.178 | 0.199 | 0.214 | 0.218 |
| Neural network (loglogistic) | 73.8 | 73.2 | 72.8 | 72.8 | 72.6 | **0.154** | 0.154 | 0.191 | 0.212 | 0.229 | 0.234 |
| Neural network (Cox) | 73.8 | **73.3** | 72.9 | 72.8 | 72.6 | 0.159 | 0.158 | 0.194 | **0.214** | 0.229 | 0.234 |
| Partial least squares | 73.8 | 73.2 | 72.8 | 72.8 | 72.6 | 0.155 | 0.109 | 0.156 | 0.189 | 0.216 | 0.228 |
| Aalen | 73.8 | 73.2 | 72.8 | 72.8 | 72.6 | **0.154** | 0.151 | 0.185 | 0.206 | 0.224 | 0.232 |
