# Supplementary material for: Optimizing predictive performance of criminal recidivism models using registration data with binary and survival outcomes
Source: PLoS One. 2019 Mar 8;14(3):e0213245. doi: 10.1371/journal.pone.0213245 (PMC6407787; doi:10.1371/journal.pone.0213245)
Supplement: S7 Table — (DOCX) [file pone.0213245.s009.docx]

**S7 Table. Predictive performance violent recidivism (survival data)**

|  | AUC  (1 yr) | AUC (2yrs) | AUC  (3 yrs) | AUC  (4 yrs) | AUC  (5 yrs) | IBS  (4 yrs) | R^2^  (1 yr) | R^2^  (2 yrs) | R^2^  (3 yrs) | R^2^  (4 yrs) | R^2^  (5 yrs) |
| --- | --- | --- | --- | --- | --- | --- | --- | --- | --- | --- | --- |
| Cox | 70.9 | 71.4 | 70.9 | 70.8 | 70.5 | 0.115 | 0.069 | 0.104 | 0.122 | 0.136 | 0.139 |
| Cox cure | 70.9 | 71.4 | 71.0 | 70.9 | 70.6 | 0.115 | 0.066 | 0.103 | 0.121 | 0.137 | 0.140 |
| Exponential | 70.9 | 71.4 | 70.9 | 70.8 | 70.5 | 0.117 | 0.045 | 0.080 | 0.104 | 0.126 | 0.135 |
| Weibull | 70.9 | 71.4 | 70.9 | 70.8 | 70.5 | 0.116 | 0.069 | 0.100 | 0.117 | 0.133 | 0.138 |
| Lognormal | 71.0 | 71.4 | 70.9 | 70.8 | 70.5 | 0.116 | 0.067 | 0.102 | 0.119 | 0.134 | 0.138 |
| Loglogistic | 70.9 | 71.4 | 70.9 | 70.8 | 70.6 | 0.115 | 0.069 | 0.102 | 0.119 | 0.134 | 0.139 |
| Cox boosting | 71.0 | 71.4 | 70.9 | 70.7 | 70.5 | 0.115 | 0.070 | 0.105 | 0.122 | 0.136 | 0.140 |
| Gradient boosting | **71.2** | **71.6** | **71.1** | **71.0** | **70.8** | **0.114** | **0.071** | **0.108** | **0.126** | **0.141** | **0.145** |
| *L*_1_-Cox | 70.9 | 71.4 | 70.9 | 70.8 | 70.5 | 0.115 | 0.069 | 0.104 | 0.122 | 0.136 | 0.139 |
| *L*_2_-Cox | 70.9 | 71.4 | 70.9 | 70.8 | 70.6 | 0.115 | 0.069 | 0.105 | 0.123 | 0.137 | 0.140 |
| Random survival forest | 68.3 | 69.2 | 68.7 | 68.7 | 68.3 | 0.181 | 0.041 | 0.075 | 0.085 | 0.096 | 0.095 |
| Neural network (exponential) | 71.0 | 71.4 | 71.0 | 70.8 | 70.6 | 0.115 | 0.067 | 0.104 | 0.122 | 0.136 | 0.139 |
| Neural network (Weibull) | 71.0 | 71.5 | 71.0 | 70.9 | 70.6 | 0.115 | 0.067 | 0.105 | 0.123 | 0.137 | 0.140 |
| Neural network (lognormal) | 71.1 | 71.5 | 71.0 | 70.9 | 70.6 | 0.115 | 0.069 | 0.105 | 0.123 | 0.137 | 0.140 |
| Neural network (loglogistic) | 71.1 | 71.5 | **71.1** | 70.9 | 70.6 | 0.115 | 0.068 | 0.105 | 0.124 | 0.138 | 0.141 |
| Neural network (Cox) | 71.1 | 71.5 | **71.1** | **71.0** | 70.7 | 0.115 | 0.068 | 0.105 | 0.124 | 0.138 | 0.141 |
| Partial least squares | 69.8 | 70.3 | 69.8 | 69.6 | 69.3 | 0.121 | 0.039 | 0.068 | 0.079 | 0.087 | 0.089 |
| Aalen | 70.6 | 71.2 | 70.8 | 70.8 | 70.5 | 0.116 | 0.063 | 0.101 | 0.119 | 0.134 | 0.137 |
