# Supplementary material for: Optimizing predictive performance of criminal recidivism models using registration data with binary and survival outcomes
Source: PLoS One. 2019 Mar 8;14(3):e0213245. doi: 10.1371/journal.pone.0213245 (PMC6407787; doi:10.1371/journal.pone.0213245)
Supplement: S8 Table — (DOCX) [file pone.0213245.s010.docx]

**S8 Table. Predictive performance sexual recidivism (survival data)**

|  | AUC  (1 yr) | AUC (2yrs) | AUC  (3 yrs) | AUC  (4 yrs) | AUC  (5 yrs) | IBS  (4 yrs) | R^2^  (1 yr) | R^2^  (2 yrs) | R^2^  (3 yrs) | R^2^  (4 yrs) | R^2^  (5 yrs) |
| --- | --- | --- | --- | --- | --- | --- | --- | --- | --- | --- | --- |
| Cox | 76.4 | 74.4 | 72.3 | 69.4 | 67.1 | 0.019 | 0.049 | 0.028 | -0.004 | -0.032 | -0.063 |
| Cox cure | 67.6 | 66.0 | 66.9 | 65.9 | 62.2 | 0.018 | -0.021 | 0.015 | 0.007 | -0.004 | -0.023 |
| Exponential | 76.2 | 73.3 | 71.3 | 68.2 | 65.9 | 0.019 | 0.106 | 0.043 | -0.007 | -0.047 | -0.086 |
| Weibull | 76.6 | 74.4 | 72.3 | 69.3 | 66.9 | 0.019 | 0.080 | 0.030 | -0.011 | -0.038 | -0.065 |
| Lognormal | 69.9 | 70.3 | 69.1 | 66.5 | 64.6 | 0.018 | 0.073 | 0.039 | 0.011 | -0.008 | -0.019 |
| Loglogistic | 76.0 | 74.6 | 72.5 | 69.4 | 67.2 | 0.018 | 0.085 | 0.043 | 0.009 | -0.016 | -0.035 |
| Cox boosting | 72.4 | 73.2 | 71.8 | 70.4 | 67.6 | 0.017 | 0.114 | 0.077 | 0.048 | 0.022 | 0.002 |
| Gradient boosting | 72.5 | 67.5 | 68.1 | 67.5 | 66.9 | 0.018 | 0.035 | 0.023 | 0.016 | 0.006 | 0.002 |
| *L*_1_-Cox | 71.7 | 67.2 | 67.8 | 65.4 | 64.0 | **0.016** | **0.172** | **0.116** | **0.098** | **0.079** | **0.070** |
| *L*_2_-Cox | 71.1 | 66.9 | 64.4 | 63.4 | 62.6 | 0.018 | 0.003 | 0.005 | 0.003 | 0.002 | 0.000 |
| Random survival forest | 63.7 | 60.8 | 50.6 | 52.5 | 50.7 | 0.048 | -2.030 | -1.226 | -1.210 | -1.651 | -1.955 |
| Neural network (exponential) | 74.2 | 69.7 | 66.3 | 63.3 | 62.2 | 0.018 | -0.004 | 0.000 | 0.000 | 0.000 | -0.002 |
| Neural network (Weibull) | 76.8 | 75.7 | 73.6 | **71.7** | **69.1** | 0.017 | 0.119 | 0.094 | 0.062 | 0.029 | 0.034 |
| Neural network (lognormal) | 72.1 | 71.4 | 69.8 | 67.3 | 65.3 | 0.017 | 0.102 | 0.082 | 0.054 | 0.026 | 0.033 |
| Neural network (loglogistic) | **77.0** | **75.9** | **73.9** | 71.6 | **69.1** | 0.017 | 0.112 | 0.092 | 0.062 | 0.030 | 0.035 |
| Neural network (Cox) | 75.5 | 74.5 | 72.7 | 70.9 | 68.4 | 0.017 | 0.110 | 0.090 | 0.060 | 0.029 | 0.032 |
| Partial least squares | 73.3 | 70.7 | 63.6 | 58.4 | 58.3 | 0.017 | 0.154 | 0.110 | 0.090 | 0.069 | 0.061 |
| Aalen | 71.3 | 69.6 | 66.2 | 63.0 | 61.0 | 0.018 | 0.043 | 0.057 | 0.051 | 0.027 | -0.016 |

* The performance random survival forests was strongly affected by the low base rate in these data. Therefore, 300% resampling of the minority class (i.e. the smallest group, namely sexual recidivists) was done for these this method. This did not substantially improve performance.
