# Supplementary material for: Optimizing predictive performance of criminal recidivism models using registration data with binary and survival outcomes
Source: PLoS One. 2019 Mar 8;14(3):e0213245. doi: 10.1371/journal.pone.0213245 (PMC6407787; doi:10.1371/journal.pone.0213245)
Supplement: S9 Table — (DOCX) [file pone.0213245.s011.docx]

**S9 Table. Predictive performance Schmidt and Witte 1978 data (survival data)**

|  | AUC  (1 yr) | AUC (2yrs) | AUC  (3 yrs) | AUC  (4 yrs) | AUC  (5 yrs) | IBS  (5 yrs) | R^2^  (1 yr) | R^2^  (2 yrs) | R^2^  (3 yrs) | R^2^  (4 yrs) | R^2^  (5 yrs) |
| --- | --- | --- | --- | --- | --- | --- | --- | --- | --- | --- | --- |
| Cox | 71.0 | 70.4 | 69.2 | 68.6 | 68.0 | 0.144 | 0.087 | 0.124 | 0.124 | 0.124 | 0.124 |
| Cox cure | 71.0 | 70.2 | 69.1 | 68.5 | 68.0 | 0.145 | 0.084 | 0.121 | 0.125 | 0.128 | 0.125 |
| Exponential | 70.2 | 69.9 | 68.6 | 68.2 | 67.6 | 0.149 | 0.055 | 0.085 | 0.085 | 0.085 | 0.085 |
| Weibull | 70.2 | 69.9 | 68.6 | 68.2 | 67.6 | 0.148 | 0.076 | 0.102 | 0.102 | 0.102 | 0.102 |
| lognormal | 70.8 | 70.1 | 68.8 | 68.3 | 67.8 | 0.147 | 0.083 | 0.110 | 0.110 | 0.110 | 0.110 |
| loglogistic | 70.7 | 70.1 | 68.8 | 68.4 | 67.8 | 0.147 | 0.083 | 0.110 | 0.110 | 0.110 | 0.110 |
| Cox boosting | 71.9 | 71.1 | 69.8 | 69.3 | 68.8 | 0.145 | 0.078 | 0.117 | 0.122 | 0.127 | 0.124 |
| Gradient boosting | **73.0** | **72.0** | **70.9** | **70.2** | **69.7** | **0.141** | **0.100** | **0.150** | **0.154** | **0.151** | **0.148** |
| *L*_1_-Cox | 71.3 | 70.7 | 69.6 | 69.1 | 68.5 | 0.144 | 0.083 | 0.126 | 0.133 | 0.135 | 0.131 |
| *L*_2_-Cox | 70.8 | 70.3 | 69.2 | 68.7 | 68.2 | 0.146 | 0.076 | 0.117 | 0.125 | 0.129 | 0.125 |
| Random survival forest | 71.2 | 69.7 | 68.9 | 67.7 | 67.0 | 0.145 | 0.092 | 0.127 | 0.129 | 0.119 | 0.112 |
| Neural network (exponential) | 71.5 | 70.5 | 69.3 | 68.7 | 68.0 | 0.145 | 0.076 | 0.124 | 0.127 | 0.126 | 0.122 |
| Neural network (Weibull) | 71.2 | 70.4 | 69.3 | 68.9 | 68.3 | 0.144 | 0.084 | 0.130 | 0.134 | 0.135 | 0.130 |
| Neural network (lognormal) | 70.6 | 69.8 | 68.7 | 68.2 | 67.7 | 0.146 | 0.077 | 0.115 | 0.123 | 0.124 | 0.122 |
| Neural network (loglogistic) | 71.6 | 70.3 | 69.6 | 69.1 | 68.5 | 0.144 | 0.087 | 0.122 | 0.136 | 0.139 | 0.134 |
| Neural network (Cox) | 71.4 | 70.3 | 69.0 | 68.6 | 67.9 | 0.144 | 0.085 | 0.136 | 0.137 | 0.138 | 0.129 |
| Partial least squares | 71.0 | 70.2 | 69.1 | 68.5 | 68.1 | 0.147 | 0.065 | 0.101 | 0.108 | 0.111 | 0.112 |
| Aalen | 70.3 | 70.0 | 69.0 | 68.3 | 67.5 | 0.146 | 0.077 | 0.113 | 0.117 | 0.120 | 0.114 |
