# Supplementary material for: Optimizing predictive performance of criminal recidivism models using registration data with binary and survival outcomes
Source: PLoS One. 2019 Mar 8;14(3):e0213245. doi: 10.1371/journal.pone.0213245 (PMC6407787; doi:10.1371/journal.pone.0213245)
Supplement: S10 Table — (DOCX) [file pone.0213245.s012.docx]

**S10 Table. Predictive performance Schmidt and Witte 1980 data (survival data)**

|  | AUC  (1 yr) | AUC (2yrs) | AUC  (3 yrs) | AUC  (4 yrs) | IBS  (4 yrs) | R^2^  (1 yr) | R^2^  (2 yrs) | R^2^  (3 yrs) | R^2^  (4 yrs) |
| --- | --- | --- | --- | --- | --- | --- | --- | --- | --- |
| Cox | 66.7 | 65.9 | 65.1 | 64.6 | 0.160 | 0.063 | 0.084 | 0.088 | 0.078 |
| Cox cure | 66.8 | 65.7 | 65.0 | 64.4 | 0.160 | 0.058 | 0.078 | 0.082 | 0.076 |
| Exponential | 66.7 | 65.8 | 65.1 | 64.5 | 0.162 | 0.042 | 0.068 | 0.080 | 0.074 |
| Weibull | 66.7 | 65.8 | 65.1 | 64.5 | 0.161 | 0.051 | 0.072 | 0.081 | 0.075 |
| Lognormal | 66.8 | 65.7 | 65.0 | 64.4 | 0.161 | 0.053 | 0.073 | 0.082 | 0.078 |
| Loglogistic | 66.9 | 65.8 | 65.1 | 64.5 | 0.161 | 0.053 | 0.075 | 0.082 | 0.076 |
| Cox boosting | 66.8 | 65.9 | 65.1 | 64.5 | 0.160 | 0.058 | 0.081 | 0.088 | 0.082 |
| Gradient boosting | **67.5** | **66.6** | **65.9** | **65.1** | **0.158** | 0.077 | **0.098** | **0.097** | 0.086 |
| *L*_1_-Cox | 67.0 | 66.1 | 65.3 | 64.7 | 0.159 | 0.062 | 0.085 | 0.089 | 0.081 |
| *L*_2_-Cox | 64.4 | 64.1 | 63.5 | 62.9 | 0.162 | 0.042 | 0.066 | 0.071 | 0.069 |
| Random survival forest | 66.6 | 65.4 | 64.7 | 63.8 | 0.161 | 0.063 | 0.080 | 0.077 | 0.067 |
| Neural network (exponential) | 66.1 | 65.3 | 64.6 | 64.0 | 0.161 | 0.056 | 0.057 | 0.057 | 0.066 |
| Neural network (Weibull) | 65.5 | 64.7 | 64.2 | 63.7 | 0.161 | 0.076 | 0.076 | 0.080 | 0.089 |
| Neural network (lognormal) | 65.6 | 65.0 | 64.6 | 64.0 | 0.160 | **0.082** | 0.080 | 0.086 | **0.094** |
| Neural network (loglogistic) | 66.8 | 65.9 | 65.4 | 64.8 | 0.159 | 0.078 | 0.078 | 0.079 | 0.088 |
| Neural network (Cox) | 66.2 | 65.4 | 64.9 | 64.3 | 0.163 | 0.030 | 0.041 | 0.042 | 0.030 |
| Partial least squares | 66.5 | 65.5 | 64.8 | 64.2 | 0.161 | 0.054 | 0.075 | 0.082 | 0.079 |
| Aalen | 66.1 | 64.9 | 63.8 | 63.5 | 0.163 | 0.051 | 0.066 | 0.064 | 0.064 |
